# Supplementary material for: A salvage pathway maintains highly functional respiratory complex I
Source: Nat Commun. 2020 Apr 2;11:1643. doi: 10.1038/s41467-020-15467-7 (PMC7118099; doi:10.1038/s41467-020-15467-7)
Supplement: Supplementary file 1 — Supplementary Information [file 41467_2020_15467_MOESM1_ESM.pdf]

Supplementary Information

A salvage pathway maintains highly functional respiratory complex I

Karolina Szczepanowska[1,2*], Katharina Senft[1,2], Juliana Heidler[3], Marija Herholz[1,2], Alexandra Kukat[1,2], Michaela Nicole Höhne[4], Eduard Hofsetz[1,2], Christina Becker[1,2], Sophie Kaspar[1,2,], Heiko Giese[5], Klaus Zwicker[6], Sergio Guerrero-Castillo[7], Linda Baumann[1,2], Johanna Kauppila[8], Anastasia Rumyantseva[1,2], Stefan Müller[1], Christian Frese[1], Ulrich Brandt[7], Jan Riemer[4], Ilka Wittig[3] and Aleksandra Trifunovic[1,2*]

Supplementary Figure 1

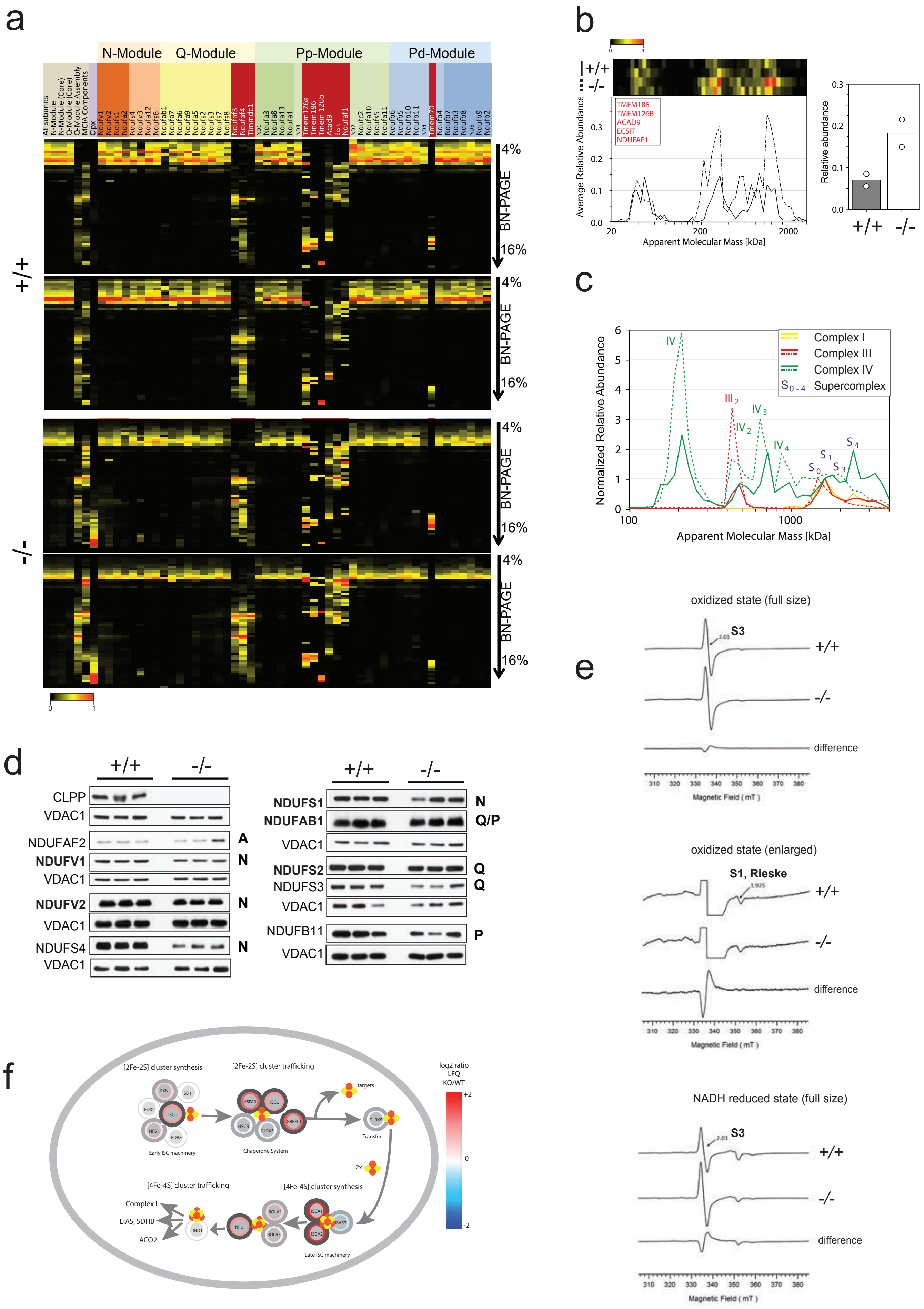

Supplementary Figure 1. Components of Complex I peripheral arm accumulate upon CLPP deficiency

(a) High-resolution complexome profiling heatmaps of the Complex I subunits and assembly factors in wild type (+/+) and CLPP deficient (-/-) heart mitochondria. (n=2, biologically independent samples).

(b) Migration profiles and corresponding quantification of the mitochondrial CI assembly factor (MCIA) complex in wild type (+/+; solid line) and CLPP deficient (-/-; dashed line) heart mitochondria. Segments of complexome profiles were subtracted from high-resolution supramolecular complexome profiling. Bars represent mean of n=2 biologically independent samples.

(c) Supramolecular organization of respiratory complexes in wild type (solid line) and CLPP deficient (dashed line) heart mitochondria. CIV (green); CIII (red); CI (yellow); supercomplexes (S_0-S_4). (n=2, biologically independent samples).

(d) Western blot analysis of the steady-state levels of individual CI subunits and assembly factors in wild type (+/+) and CLPP deficient (-/-) heart mitochondria. VDAC1 was used as a loading control. CLPP substrates are shown in bold. A: assembly factor; N: N-module; Q: Q-module; P: membrane arm. (n=3, biologically independent samples).

(e) EPR spectroscopy of mitochondrial membranes from wild type (+/+) and CLPP deficient (-/-) hearts in the oxidized state (*top* and *middle panel*). Characteristic g-values, 2.01 for cluster S3 and 1.925, most likely due to some reduced cluster S1 or Rieske cluster are indicated. The difference spectrum does not reveal any striking differences in EPR signals except a slightly higher content of oxidized cluster S3 in the mutant. (Spectra were normalized to same protein concentration; 10 scans accumulated for each spectrum). *Bottom panel:* the EPR spectra of mitochondrial membranes upon reduction by NADH. Signals from complex I Fe-S clusters can be identified at the low and high field site of the predominant S3 signal (g = 2.01).

(f) Schematic mapping of the proteome changes associated with the cardiac CLPP deficiency in a pathway of biosynthesis, assembly and trafficking of iron-sulfur clusters. Data collected from the label-free quantitative proteome analysis from wild type (WT) and CLPP deficient (KO) heart mitochondria.

Supplementary Figure 2

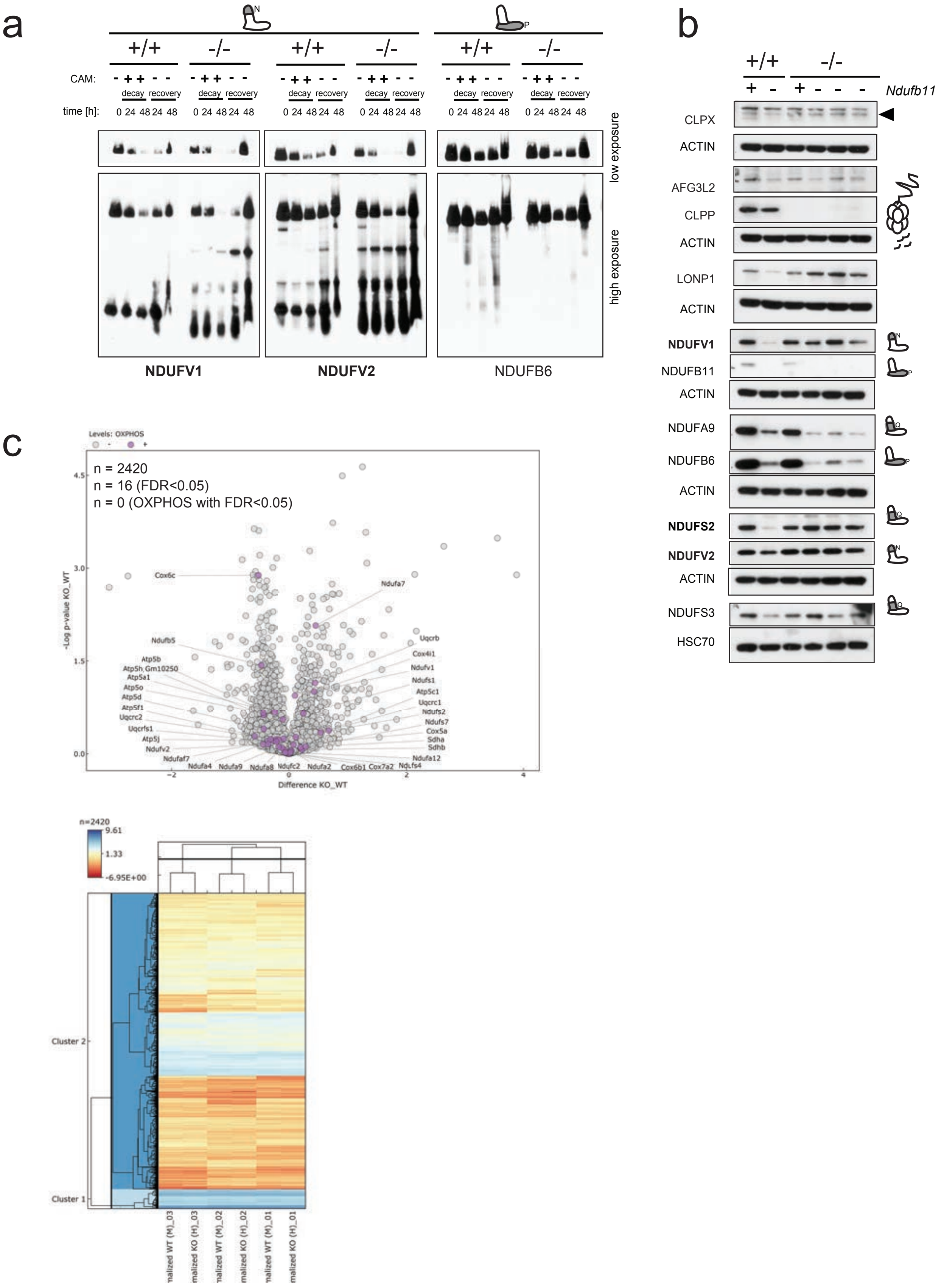

Supplementary Figure 2. ClpXP protease regulates the turnover of N-module

(a) Time-lapse BN-PAGE followed by Western blot analysis of CI profiles in wild type (+/+) and CLPP deficient (-/-) MEFs upon chloramphenicol (CAM)-mediated decay of respiratory chain complexes and subsequent recovery of the mitochondrial protein synthesis resulting in *de novo* assembly of mitochondrial respiratory complexes (n=2, biologically independent experiments).

(b) Western blot analysis of the steady-state levels of individual CI subunits and protein quality control components in wild type (*Clpp*$^{+/+}$) and CLPP deficient (*Clpp*$^{-/-}$) cells upon CRISPR/Cas9 depletion of NDUFB11 subunit. Three biologically independent clones were used for double-deficient cells.

(a-b) Antibodies used were raised against proteins indicated in the Figure, with putative CLPP substrates shown in bold. ß-actin and HSC70 were used as loading controls.

(c) Quantitative analysis of the global *de novo* protein synthesis in wild type (WT) and CLPP deficient (KO) MEFs. Cells were briefly pulsed labelled (30 minutes) with medium (WT) or heavy (KO) SILAC in the presence of methionine analog, L-azidohomoalanine (L-AHA). Newly synthesized peptides were enriched via click-chemistry followed by their identification and quantification by mass spectrometry. Data are shown in volcano plots (*top panel*). None of the OXPHOS subunits accomplishes the FDR<0.05 limits. Violet represent the OXPHOS subunits. *Bottom panel*: clustering heatmap for the log2 protein intensities of each individual sample. (n=3, biologically independent samples).

Supplementary Figure 3

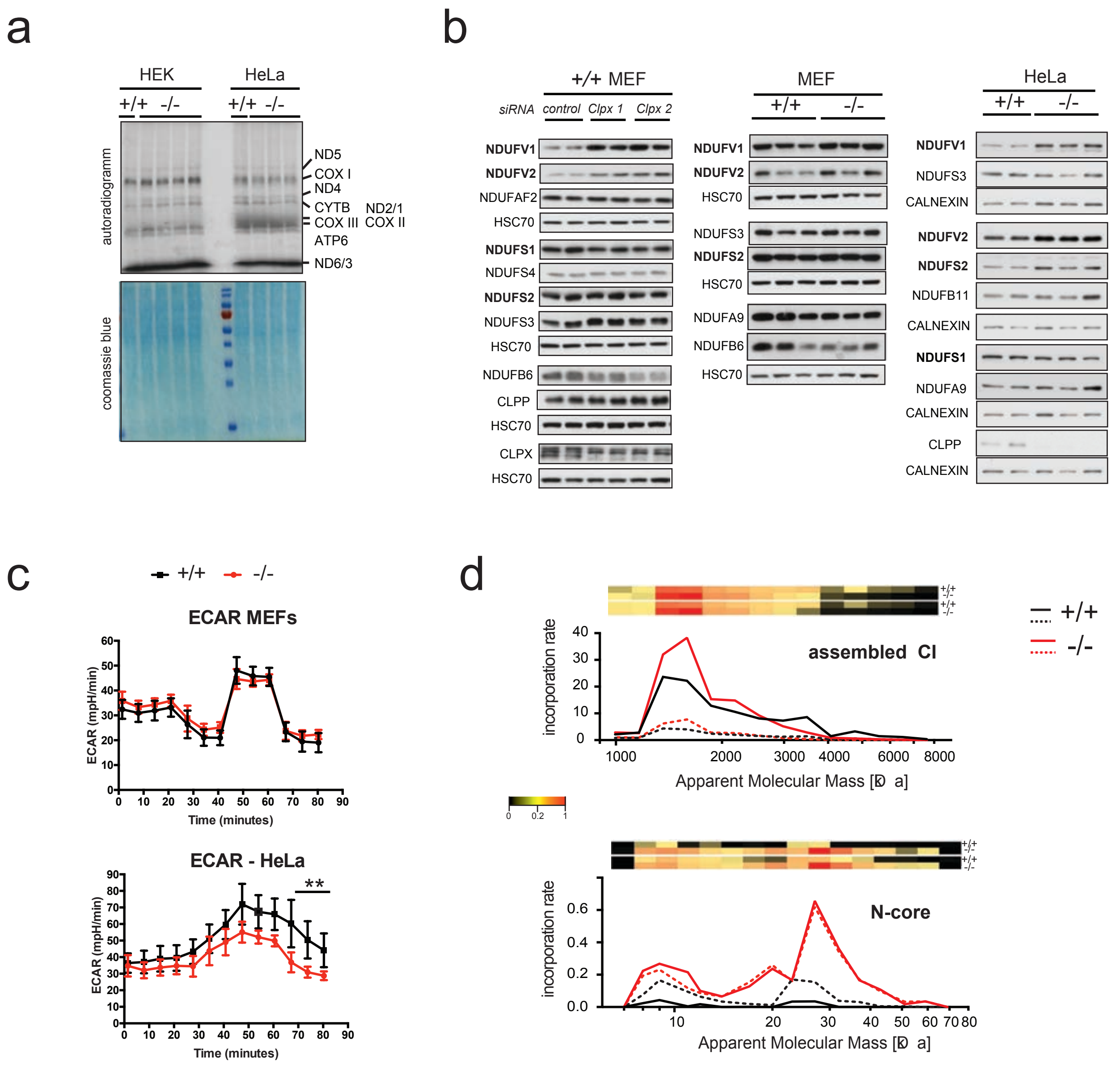

Supplementary Figure 3. Accumulation of modified N-module causes respiratory chain deficiency

(a) *De novo* synthesis of mitochondrial proteins in wild type (+/+) and CLPP deficient (-/-) HEK 293t and HeLa cells followed by SDS-PAGE. Coomassie blue staining was used as a loading control. Individual lanes represent independent clones.

(b) Western blot analysis of the steady-state levels of individual Complex I subunits in: *left panel* - wild type (+/+) MEFs upon the siRNA-mediated knockdown of CLPX using two independent siRNA probes; *middle panel* - wild type (+/+) and CLPP deficient (-/-) MEFs; *right panel* CRIPSR/Cas9 engineered CLPP knockout HELA cells (-/-). HSC70 and calnexin were used as loading controls. Individual lanes represent independent clones. (n=2, biologically independent experiments)

(c) Extracellular Acidification Rates (ECAR) in wild type (+/+) and CLPP deficient (-/-) MEF and HeLa cells corresponding to the OCR presented on a Figure 3B. Error bars represent means ± SD. (MEFs n=5-6; HeLa n=6, biologically independent samples).

(d) Steady state (solid line) and incorporation rates (dashed line) of CI subunits in wild type (+/+; black) and CLPP deficient (-/-; red) MEFs. *Upper panel* Assembled CI at the level of supercomplexes (fractions 7628-995); *Lower panel* Core N-module subunits (NDUFS1, NDUFV1 and NDUFV2) at the level of free N-module subcomplexes (fractions 69-7). Detailed analysis is described in Supplementary Data 5

Supplementary Figure 4

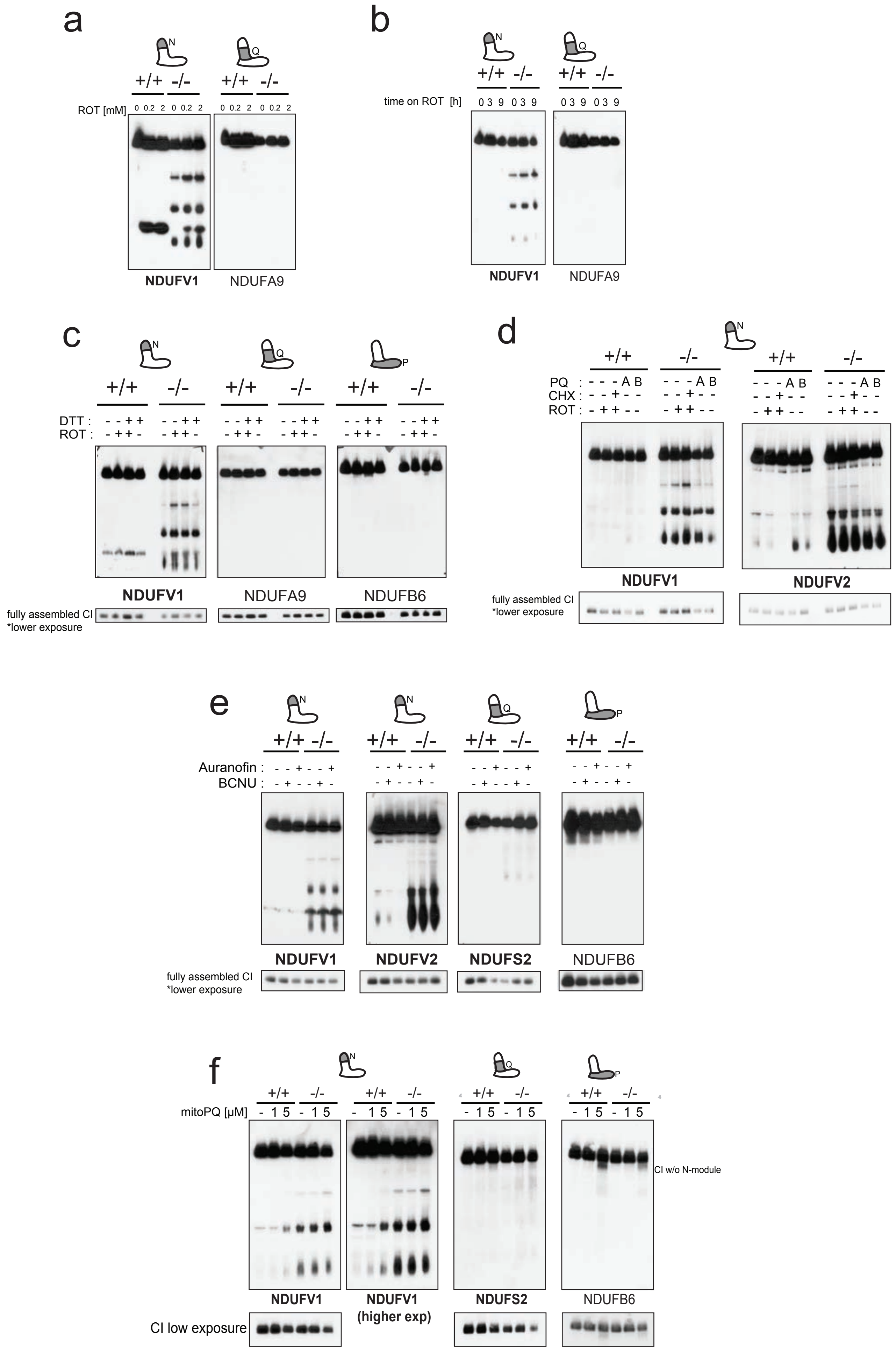

Supplementary Figure 4. Oxidative stress dictates the N-module turnover.

(a-e) BN-PAGE followed by Western blot analysis of CI in wild type (+/+) and CLPP deficient (-/-) MEFs. Antibodies used were raised against proteins indicated in the Figure, with putative CLPP substrates shown in bold. Cells were treated: **(a)** 8h with increasing rotenone (ROT) concentrations; **(b)** 200 μM rotenone (ROT) for different periods of time; **(c)** 8h in the presence of rotenone (1 mM) and/or DTT (0.5 mM); **(d)** 10h with rotenone (ROT; 10 μM) and/or cycloheximide (CHX; 100 μM), and paraquat (PQ; A: 0.25 mM, B: 1 mM) **(e)** 2h with BCNU (10 μM) or auranofin (1μM); **(f)** 12h with increasing mito-paraquat (mitoPQ) concentrations. (n=2, biologically independent experiments).

Supplementary Figure 5

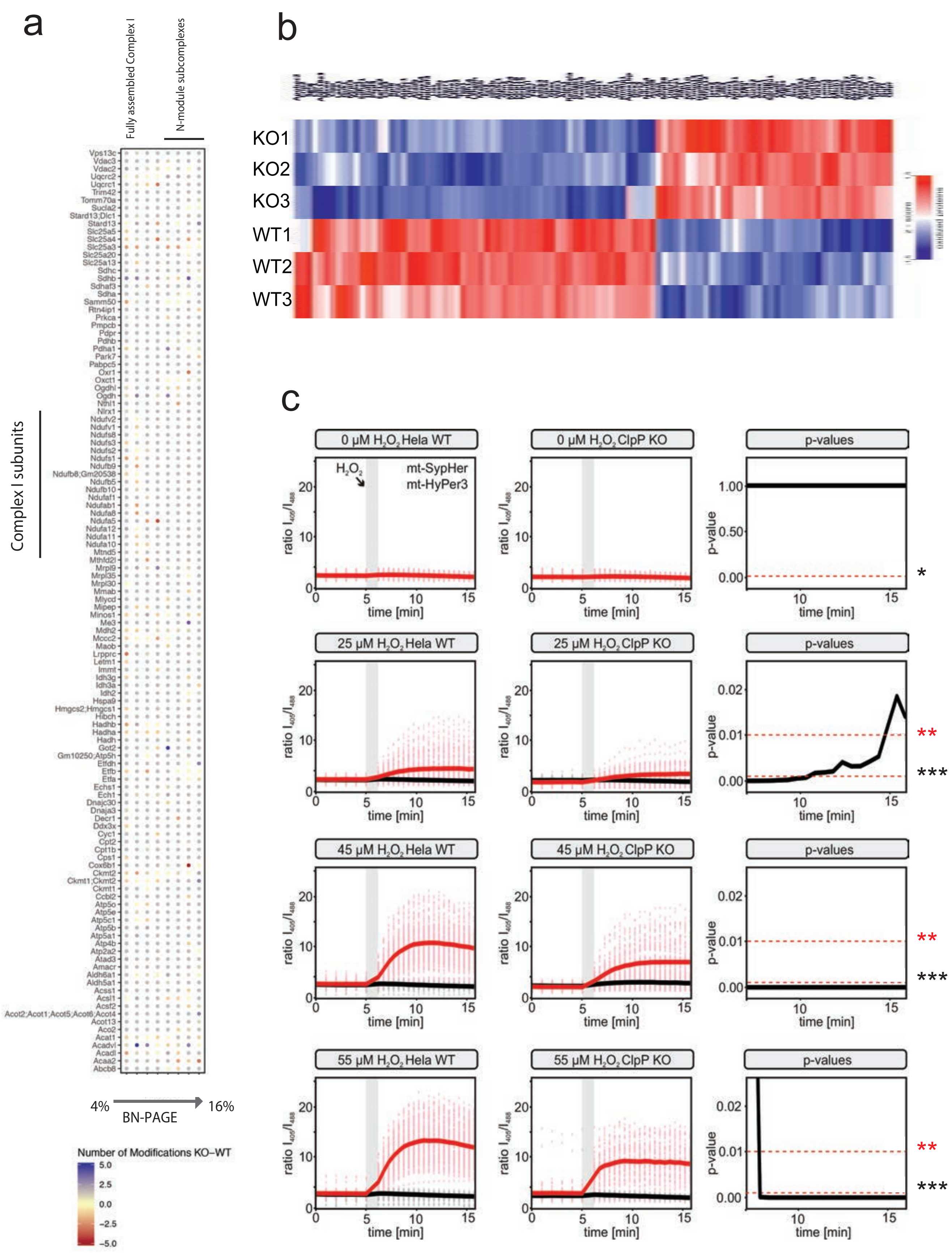

Supplementary Figure 5. CLPP deficiency prevents from oxidative damage.

(a) Global analysis of the protein oxidative damage detected on peptides analysed with quantitative low-resolution complexome profiling. Thiol modification was not performed prior to the mass spectrometry, therefore only irreversible oxidative changes can be faithfully determined with this analysis. (n=3, biologically independent samples).

(b) Heatmap of thiol-oxidation in mitochondrial proteins of wild type (WT) and CLPP deficient (KO) mouse hearts. Z-Score of significant oxidized proteins normalized to protein abundance (FDR>0.05, n=3, biologically independent samples).

(c) Raw data representation of the ratiometric imaging for response to hydrogen peroxide of Hela wild type and ClpP knock out cells, as presented in Figure 5h. Cells containing the matrix-targeted H2O2 sensor HyPer3 or as pH-control matrix-targeted SypHer sensors were analysed for their response towards 45 µM hydrogen peroxide. While at steady state both cell lines exhibit similar ratios for the HyPer3 sensor, the addition of hydrogen peroxide results in an increased response for HeLa wild type (+/+) cells compared to HeLa ClpP knockout cells (-/-). The SypHer sensor was not deflected during the experiment indicating that the HyPer3 response was solely due to changes in hydrogen peroxide levels. Results were from two independent experiments (n[HeLa, HyPer3]= 141 cells, n[ClpP KO, HyPer3]= 70 cells, n[HeLa, SypHer]= 89 cells, n[ClpP KO, SypHer]= 75 cells. Shapiro-Wilk test to test for normal distribution followed by Mann-Whitney-U test was used to determine the level of statistical difference presented by asteriks (*p<0.05, **p<0.01; ***p<0.001).

Supplementary Figure 6

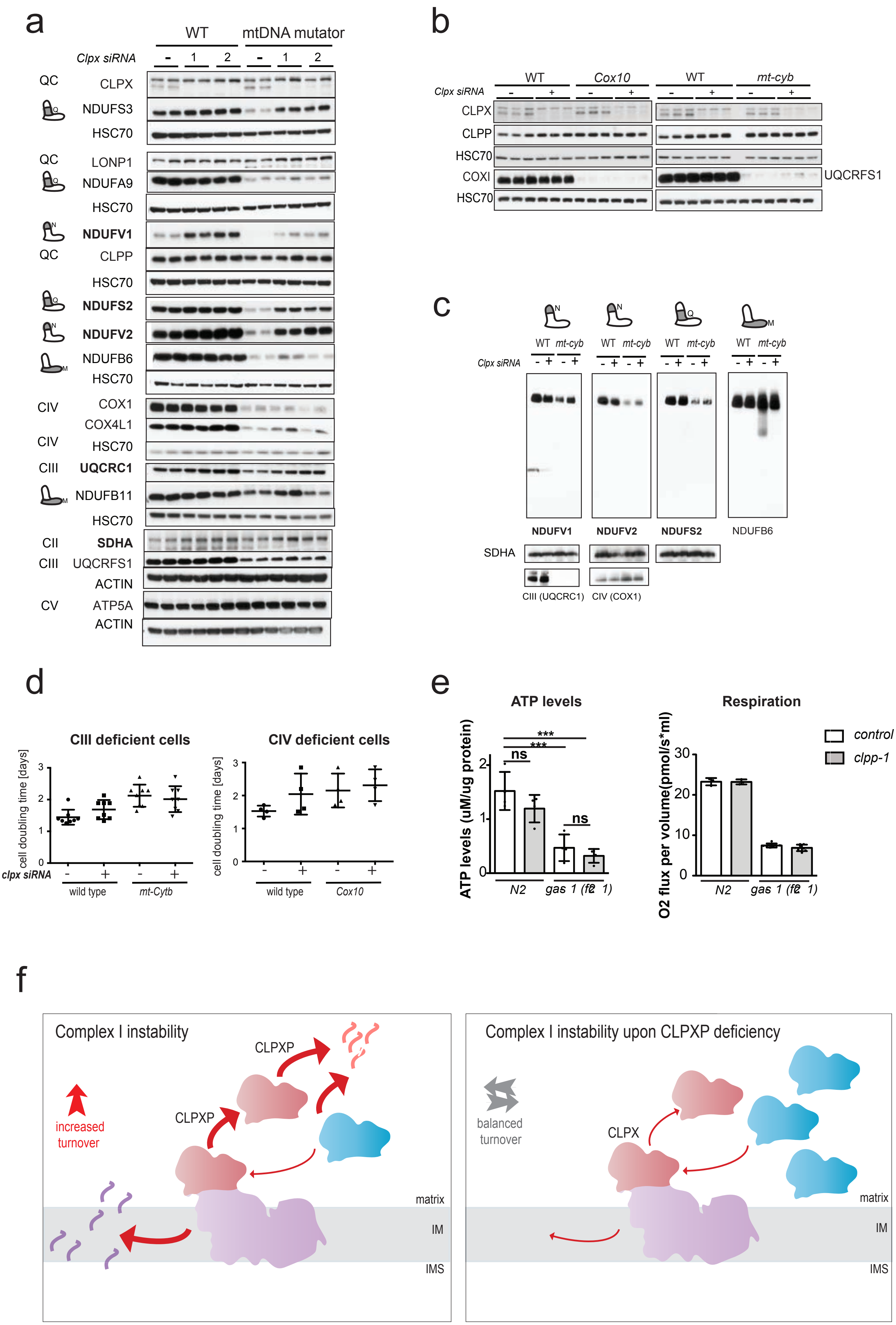

Supplementary Figure 6. Loss of CLPXP ameliorates phenotypes caused by MRC instability

(a) Western blot analysis of the steady-state levels of individual OXPHOS subunits and protein quality control (QC) components in wild type (WT) and *PolgA*^{mut}/^{mut} (mtDNA mutator) MEFs upon the 48 hours *siRNA*-mediated *Clpx* knockdown (using two independent siRNA probes). HSC70 and beta-actin were used as loading controls. CLPP substrates are shown in bold. A: assembly factor; N: N-module; Q: Q-module; P: membrane arm. Individual lanes represent independent biological samples.

(b) Western blot analysis of the levels of CLPX, CLPP and subunits of respiratory complexes in CIV-deficient COX10 fibroblasts (*cox10*), cytochrome b mutant (*mt-cyb*) cybrids, and respective wild type controls (WT) upon the 48 hours *siRNA*-mediated *Clpx* knockdown. This panel was used as a control for the Figure 6 c, Supplementary Figure 6 c and 6 d. HSC70 was used as a loading control (n=3; independent biological samples).

(c) BN-PAGE followed by Western blot analysis of CI in wild type (WT) and cytochrome b mutant (*mt-cyb*) cybrids upon the 48 hours siRNA-mediated CLPX knockdown. Antibodies used were raised against proteins indicated in the Figure, with putative CLPP substrates shown in bold. (n=2, independent biological experiments).

(d) Proliferation capacity of the cytochrome b mutant (*mt-cyb*) cybrids, the CIV-deficient COX10 fibroblasts (*cox10*) and respective wild types upon the 48 hours *siRNA*-mediated *Clpx* knockdown assessed 48 hours after transfection and estimated as a cell doubling time. Control cells were transfected with control siRNA. Bars represent mean ± SD. Two-way ANOVA and Sidak's post test were used to determine the level of statistical difference (n=4-8, independent biological samples)

(e) *Left panel:* ATP levels in D1 adult wild type N2 worms and *gas-1* mutant grown on control L4440 (EV) or *clpp-1* RNAi plates. Data is normalized to the total amount of protein. One-way ANOVA with Tukey post hoc test. (n=4, independent biological samples). *Right panel:* Respiration measurements of D1 adult wild type N2 and *gas-1* mutant raised on either control L4440 (EV) or *clpp-1* RNAi plates. One-way ANOVA with Tukey post hoc test was used to determine the level of statistical difference. (n=6, independent biological samples).

(f) A comprehensive model of the ClpXP-mediated surveillance of CI in mitochondrial disease models of OXPHOS instability.
