# Supplementary material for: A salvage pathway maintains highly functional respiratory complex I
Source: Nat Commun. 2020 Apr 2;11:1643. doi: 10.1038/s41467-020-15467-7 (PMC7118099; doi:10.1038/s41467-020-15467-7)
Supplement: Supplementary file 3 — Description of Additional Supplementary Files [file 41467_2020_15467_MOESM3_ESM.pdf]

Description of Additional Supplementary Files

File Name: Supplementary Data 1

Description: pSILAC turnover in C2C12

File Name: Supplementary Data 2

Description: HR Complexomics heart

File Name: Supplementary Data 3

Description: LFQ heart mitochondria

File Name: Supplementary Data 4

Description: pSILAC-AHA enrichment for newly synthesized proteins

File Name: Supplementary Data 5

Description: pSILAC turnover in WT and CLPP KO MEFs

File Name: Supplementary Data 6

Description: CI in WT and TRAP OE MEFs

File Name: Supplementary Data 7

Description: oxidative damage in LR Complexomics heart/LR Complexomics

File Name: Supplementary Data 8

Description: BIAM assay heart
