# Supplementary material for: A salvage pathway maintains highly functional respiratory complex I
Source: Nat Commun. 2020 Apr 2;11:1643. doi: 10.1038/s41467-020-15467-7 (PMC7118099; doi:10.1038/s41467-020-15467-7)
Supplement: Supplementary file 12 — Reporting Summary [file 41467_2020_15467_MOESM12_ESM.pdf]

nature research

Corresponding author(s): Aleksandra Trifunovic

Last updated by author(s): March 5th 2020

Reporting Summary

Nature Research wishes to improve the reproducibility of the work that we publish. This form provides structure for consistency and transparency in reporting. For further information on Nature Research policies, see Authors & Referees and the Editorial Policy Checklist .

Statistics

For all statistical analyses, confirm that the following items are present in the figure legend, table legend, main text, or Methods section.

n/a	Confirmed	
☐	☒	The exact sample size (*n*) for each experimental group/condition, given as a discrete number and unit of measurement
☐	☒	A statement on whether measurements were taken from distinct samples or whether the same sample was measured repeatedly
☐	☒	The statistical test(s) used AND whether they are one- or two-sided<br>*Only common tests should be described solely by name; describe more complex techniques in the Methods section.*
☒	☐	A description of all covariates tested
☐	☒	A description of any assumptions or corrections, such as tests of normality and adjustment for multiple comparisons
☐	☒	A full description of the statistical parameters including central tendency (e.g. means) or other basic estimates (e.g. regression coefficient) AND variation (e.g. standard deviation) or associated estimates of uncertainty (e.g. confidence intervals)
☐	☒	For null hypothesis testing, the test statistic (e.g. *F*, *t*, *r*) with confidence intervals, effect sizes, degrees of freedom and *P* value noted<br>*Give P values as exact values whenever suitable.*
☒	☐	For Bayesian analysis, information on the choice of priors and Markov chain Monte Carlo settings
☒	☐	For hierarchical and complex designs, identification of the appropriate level for tests and full reporting of outcomes
☒	☐	Estimates of effect sizes (e.g. Cohen's *d*, Pearson's *r*), indicating how they were calculated

Our web collection on statistics for biologists contains articles on many of the points above.

Software and code

Policy information about availability of computer code

Data collection	All mass spectrometry proteomic data have been provided in a form of Excel spreadsheets. Mass spectrometry proteomics data have also been deposited to the ProteomeXchange Consortium via the PRIDE partner repository with the following dataset identifiers:<br><br>PXD014897 (http://www.ebi.ac.uk/pride/archive/projects/PXD014897);<br>PXD017463 (https://www.ebi.ac.uk/pride/archive/projects/PXD017463);<br>PXD017464 (http://www.ebi.ac.uk/pride/archive/projects/PXD017464);<br>PXD017465 (http://www.ebi.ac.uk/pride/archive/projects/PXD017465);<br>PXD017614 (https://www.ebi.ac.uk/pride/archive/projects/PXD017614).

Data analysis

ImageJ 1.43
Seahorse Wave Desktop Software
Excel 2016
Excel 2013
GraphPrism 5
MACS2 version 2.0.10
Maxquant 1.5.3.8
Maxquantv1.6.0.1
Perseus 1.5.6.0
Instant Clue for Mac
RRA ("redox ratio analysis")

For manuscripts utilizing custom algorithms or software that are central to the research but not yet described in published literature, software must be made available to editors/reviewers. We strongly encourage code deposition in a community repository (e.g. GitHub). See the Nature Research guidelines for submitting code & software for further information.

Data

Policy information about availability of data

All manuscripts must include a data availability statement. This statement should provide the following information, where applicable:

- Accession codes, unique identifiers, or web links for publicly available datasets
- A list of figures that have associated raw data
- A description of any restrictions on data availability

The source data underlying this study are provided as a Source Data file or available from the authors upon request.
List of figures with associated raw data has been provided.
All mass spectrometry proteomic data have been provided in a form of Excel spreadsheets. Furthermore, part of those mass spectrometry proteomics data have been deposited to the ProteomeXchange Consortium via the PRIDE partner repository with the dataset identifier PXD014897. Editors and Reviewers may access these data with following accounts details:

Project accession: PXD014897.
Username: reviewer55053@ebi.ac.uk
Password: I8pTu019

Project accession: PXD017465
Username: reviewer08382@ebi.ac.uk
Password: u7T3GiK4

Project accession: PXD017464
Username: reviewer35612@ebi.ac.uk
Password: FLdwrjIm

Project accession: PXD017463
Username: reviewer44818@ebi.ac.uk
Password: 7p5TAsQh

Field-specific reporting

Please select the one below that is the best fit for your research. If you are not sure, read the appropriate sections before making your selection.

☒ Life sciences ☐ Behavioural & social sciences ☐ Ecological, evolutionary & environmental sciences

For a reference copy of the document with all sections, see nature.com/documents/nr-reporting-summary-flat.pdf

Life sciences study design

All studies must disclose on these points even when the disclosure is negative.

Sample size	Sample sizes were variable and depended on the type of experiment and model organism (mice, cultured cells, worms). All sample sizes were annotated within the respective Figure legends.
Data exclusions	No data was excluded
Replication	For analysis of animal samples: Independent animals were used for each experimental replicate. Due to material requirements, for some experiments (EPR, import assay, IP) several animals with identical genotype have been pooled prior to the analysis.<br>For cell culture experiments: Biological replicates of particular cell line, or several independently selected clones of each genotype were used across the study. Type and number of replicates was indicated in respective figure legend if applicable.

	For analysis of worms: 3 replicates
Randomization	For animal experiments: if possible, littermates of opposite genotypes were analyzed together. Animals were age-matched. Animals were not sex-matched.<br>For cell culture experiments: different clones were experimentally tested if available.<br>For roundworm experiments: for all experiments, worms were randomly selected
Blinding	No blinding was applied.

Reporting for specific materials, systems and methods

We require information from authors about some types of materials, experimental systems and methods used in many studies. Here, indicate whether each material, system or method listed is relevant to your study. If you are not sure if a list item applies to your research, read the appropriate section before selecting a response.

Materials & experimental systems

n/a	Involved in the study
☐	☒ Antibodies
☐	☒ Eukaryotic cell lines
☒	☐ Palaeontology
☐	☒ Animals and other organisms
☒	☐ Human research participants
☒	☐ Clinical data

Methods

n/a	Involved in the study
☒	☐ ChIP-seq
☒	☐ Flow cytometry
☒	☐ MRI-based neuroimaging

Antibodies

Antibodies used	NDUFV2 (Proteintech #15301-1-AP); NDUFV1 (Proteintech #11238-1-AP); NDUFS1 (Proteintech #12444-1-AP); NDUFS2 (Abcam #ab96160); NDUFS3 (MitoSciences #MS112); NDUFA9 (Molecular Probes #459100); NDUFB11 (Abcam #ab183716); NDUFAF2 (Abcam #ab192267); NDUFS4 (Abcam #ab87399); NDUFAB1 (Abcam #ab96230); NDUFB6 (Invitrogen #A21359); SDHA (Molecular Probes #459200); MT-CO1 (Molecular Probes #459600); COX4L1 (Molecular Probes #A21348); UQCRC1 (Molecular Probes #459140); UQCRFS1 (MitoSciences #MS305); ATP5A (Abcam #ab14748); ACO2 (Abcam #ab110321); CLPP (Sigma #10008192M1-100); CLPX (Sigma #HPA040262); LONP1 (Abcam #ab103809); AFG3L2 (a kind gift from Prof. E.I. Rugarli, University of Cologne); VDAC (Cell Signaling #4661); β-ACTIN (Sigma #A5441); HSC70 (Santa Cruz #sc7298); CALNEXIN (Calbiochem #208880)
Validation	Antibodies were validated by manufacturer and in this study. Generally, data obtained with immunoblotting highly matched the data obtained with mass spectrometry proteomics.

Eukaryotic cell lines

Policy information about cell lines

Cell line source(s)	CLPP deficient mouse embryonic fibroblasts were isolated and immortalized in the lab of Aleksandra Trifunovic.<br>mtDNA mutator mouse embryonic fibroblasts (PolgD257A/D257A) were isolated and immortalized in a lab of Aleksandra Trifunovic<br>NDUFB11 and NDUFB11/CLPP double deficient mouse embryonic fibroblasts were generated with Crisp/cas9 in a lab of Aleksandra Trifunovic during the course of this project.<br>HEK293T cells were from ATCC<br>CLPP deficient HEK293T cells were generated with Crisp/cas9 in a lab of Aleksandra Trifunovic<br>HeLa cells were from ATCC<br>CLPP deficient HeLa cells were generated with Crisp/cas9 in a lab of Aleksandra Trifunovic<br>L929 Cytb mutant and control cells were a kind gift from Jose Antonio Enriquez<br>Cox10 KO and control cells were a kind gift from Jose Antonio Enriquez
Authentication	HEK293T and HeLa cells have been authenticated by ATCC<br>Cells generated in house were validated by genotyping, western-blotting and/or sequencing
Mycoplasma contamination	Negative for mycoplasma contamination
Commonly misidentified lines<br>(See ICLAC register)	No commonly misidentified lines were used.

Animals and other organisms

Policy information about studies involving animals; ARRIVE guidelines recommended for reporting animal research

Laboratory animals	mice, Mus musculus, C57BL/6 NTac, mixed sex, age 18-24 weeks
Wild animals	*Provide details on animals observed in or captured in the field; report species, sex and age where possible. Describe how animals were caught and transported and what happened to captive animals after the study (if killed, explain why and describe method; if released, say where and when) OR state that the study did not involve wild animals.*
Field-collected samples	*For laboratory work with field-collected samples, describe all relevant parameters such as housing, maintenance, temperature, photoperiod and end-of-experiment protocol OR state that the study did not involve samples collected from the field.*
Ethics oversight	All experiments were approved and permitted by the Animal Ethics Committee of North-Rhein Westphalia (Landesamt für Natur, Umwelt und Verbraucherschutz Nordrhein-Westfalen; LANUV) following the German and European Union regulations. All animal work was performed in accordance with recommendations and guidelines of the Federation of European Laboratory Animal Science Associations (FELASA).

Note that full information on the approval of the study protocol must also be provided in the manuscript.
