# Supplementary material for: A salvage pathway maintains highly functional respiratory complex I
Source: Nat Commun. 2020 Apr 2;11:1643. doi: 10.1038/s41467-020-15467-7 (PMC7118099; doi:10.1038/s41467-020-15467-7)

Figure 1 Panel B

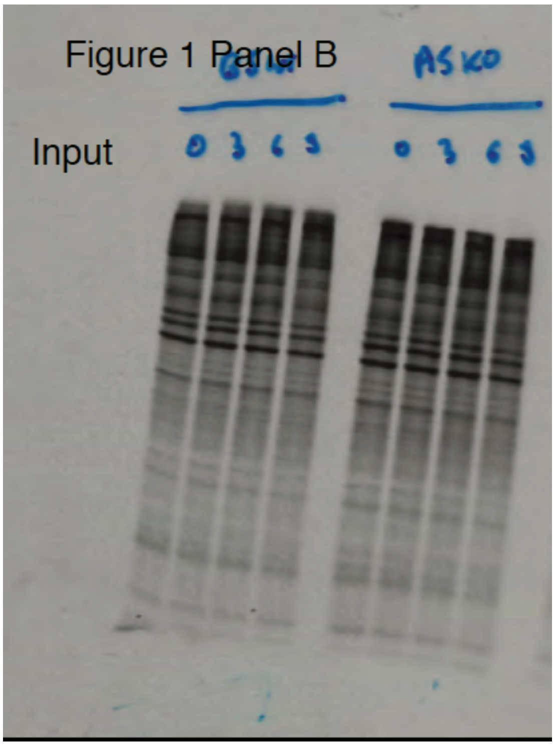

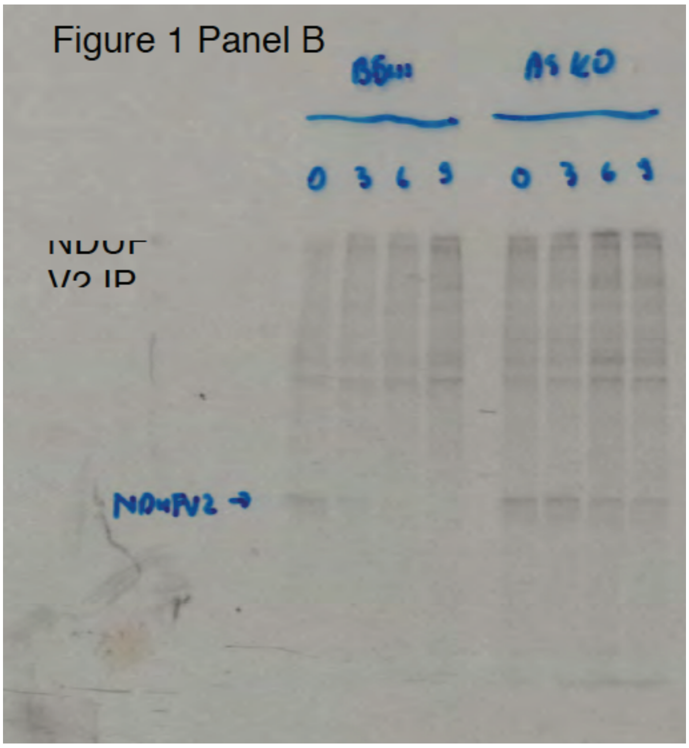

Figure 1 Panel C

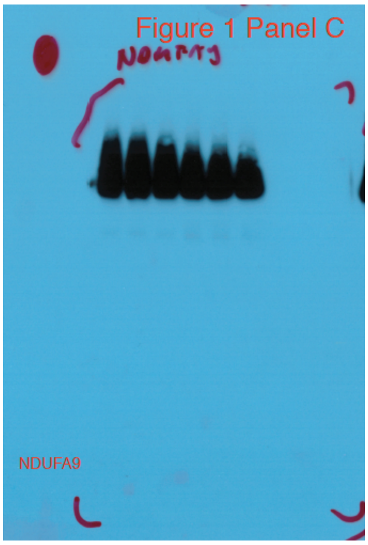

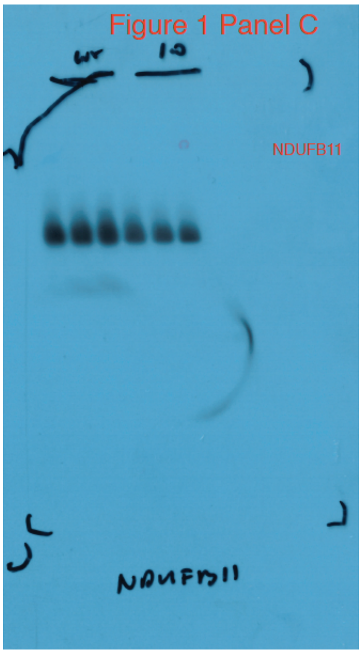

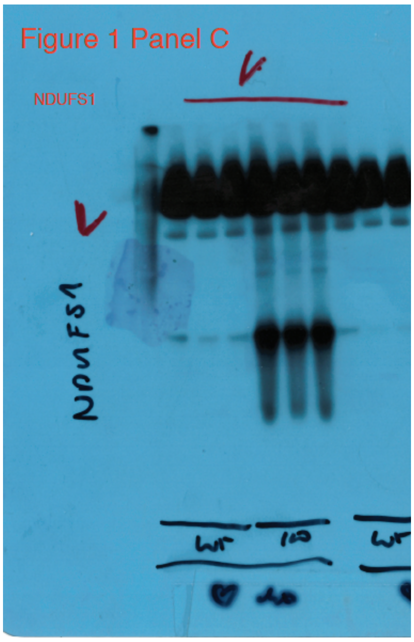

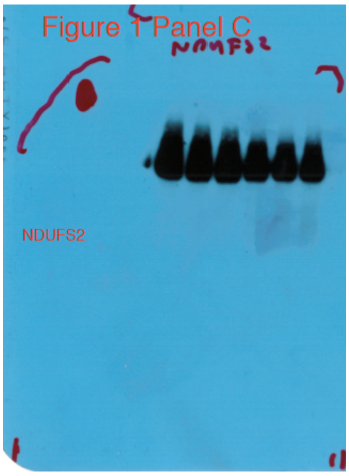

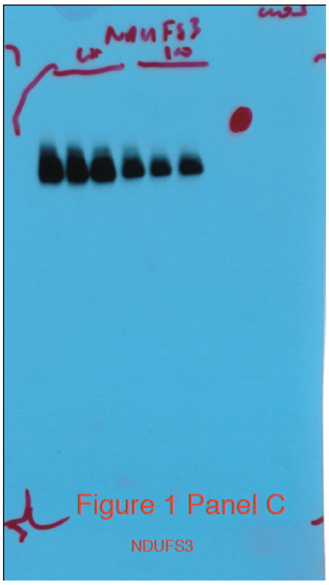

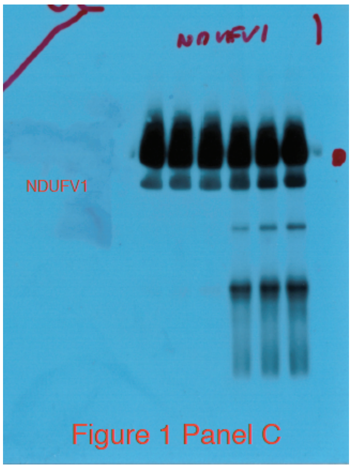

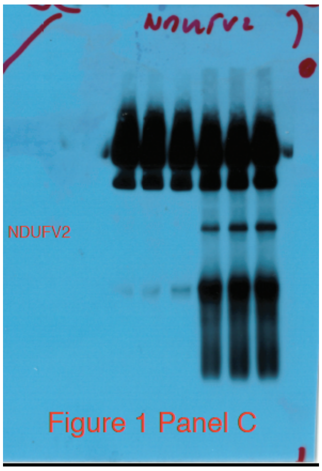

Figure 2 Panel A

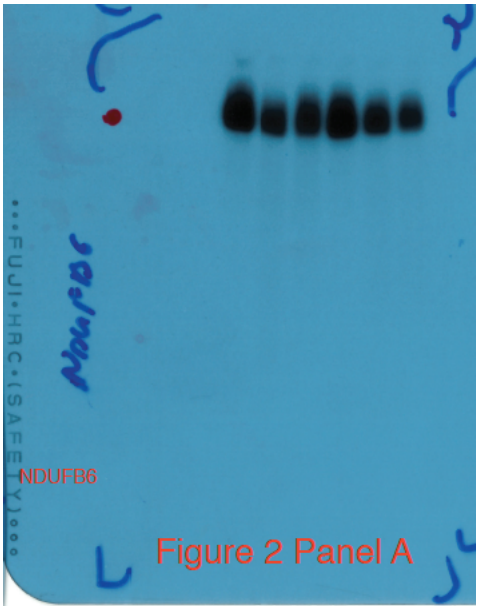

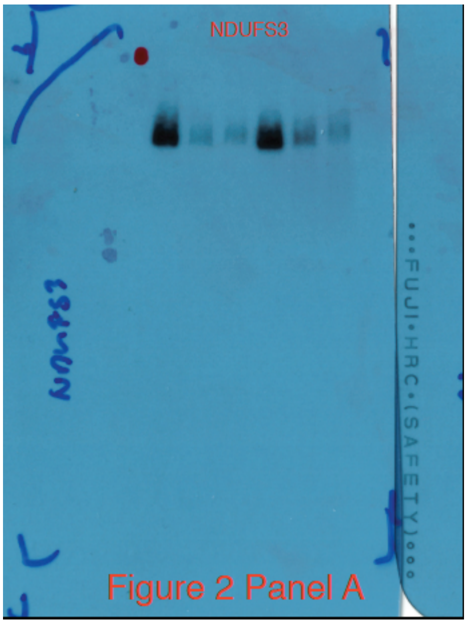

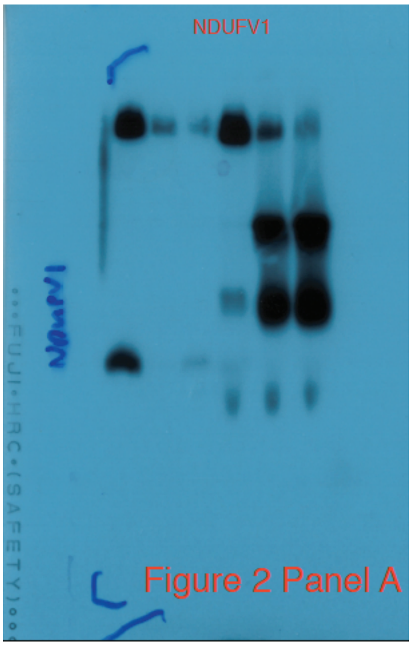

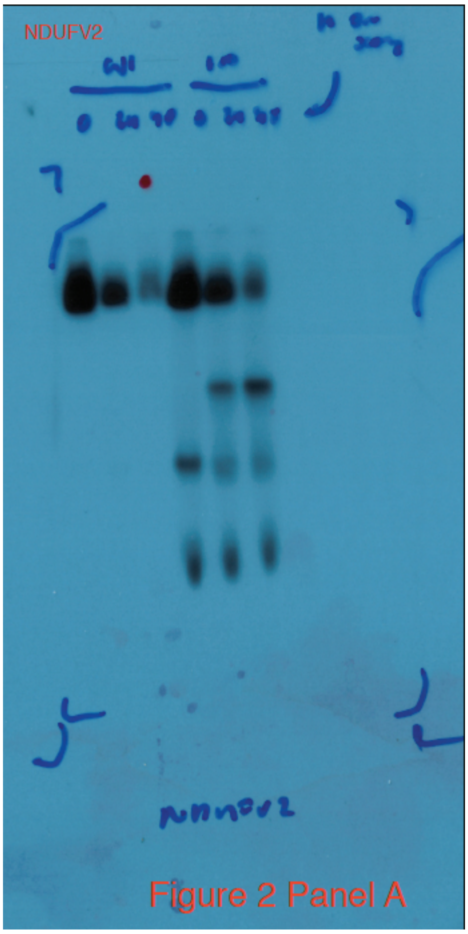

Figure 2 Panel B

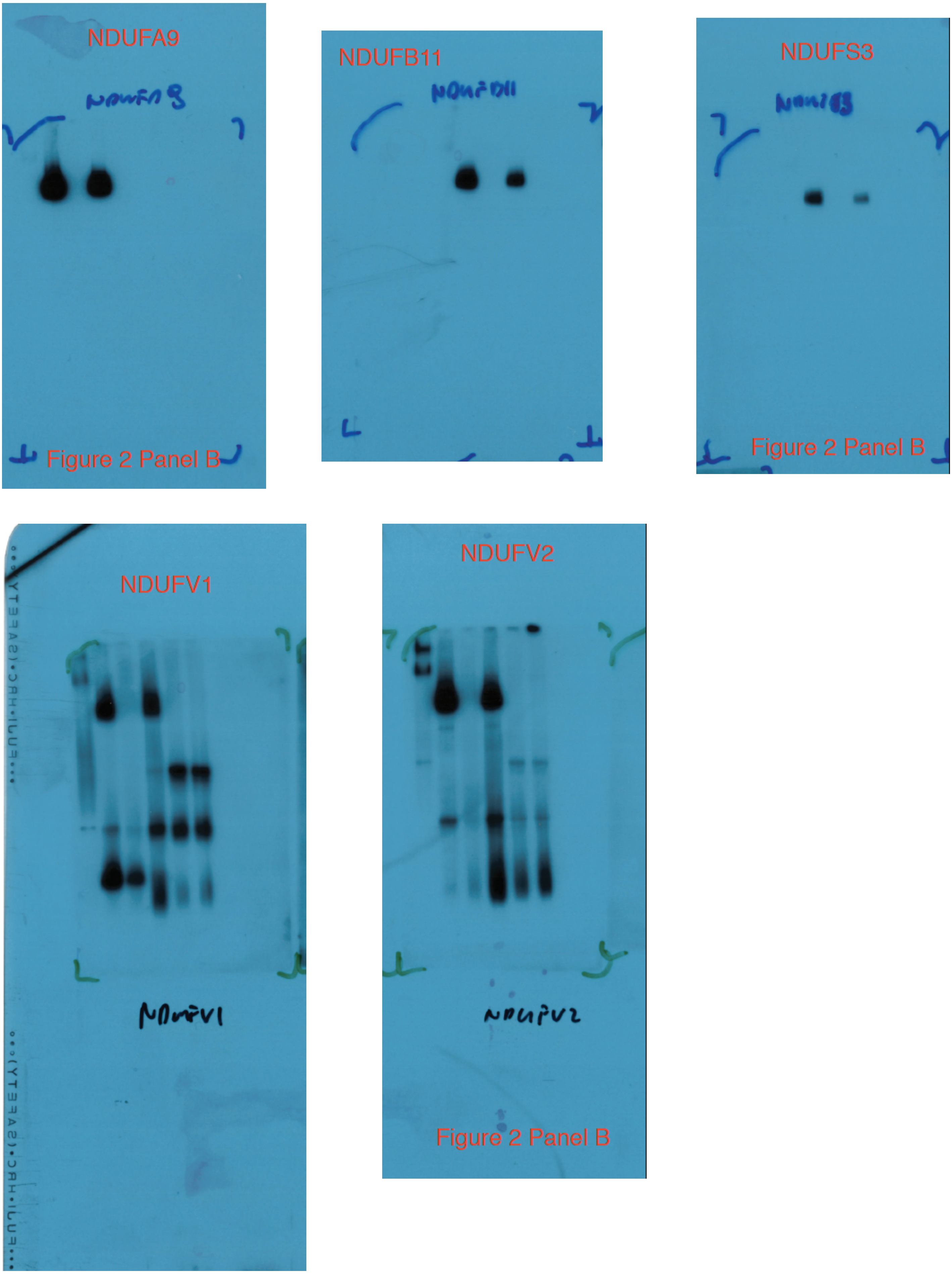

Figure 2 Panel C

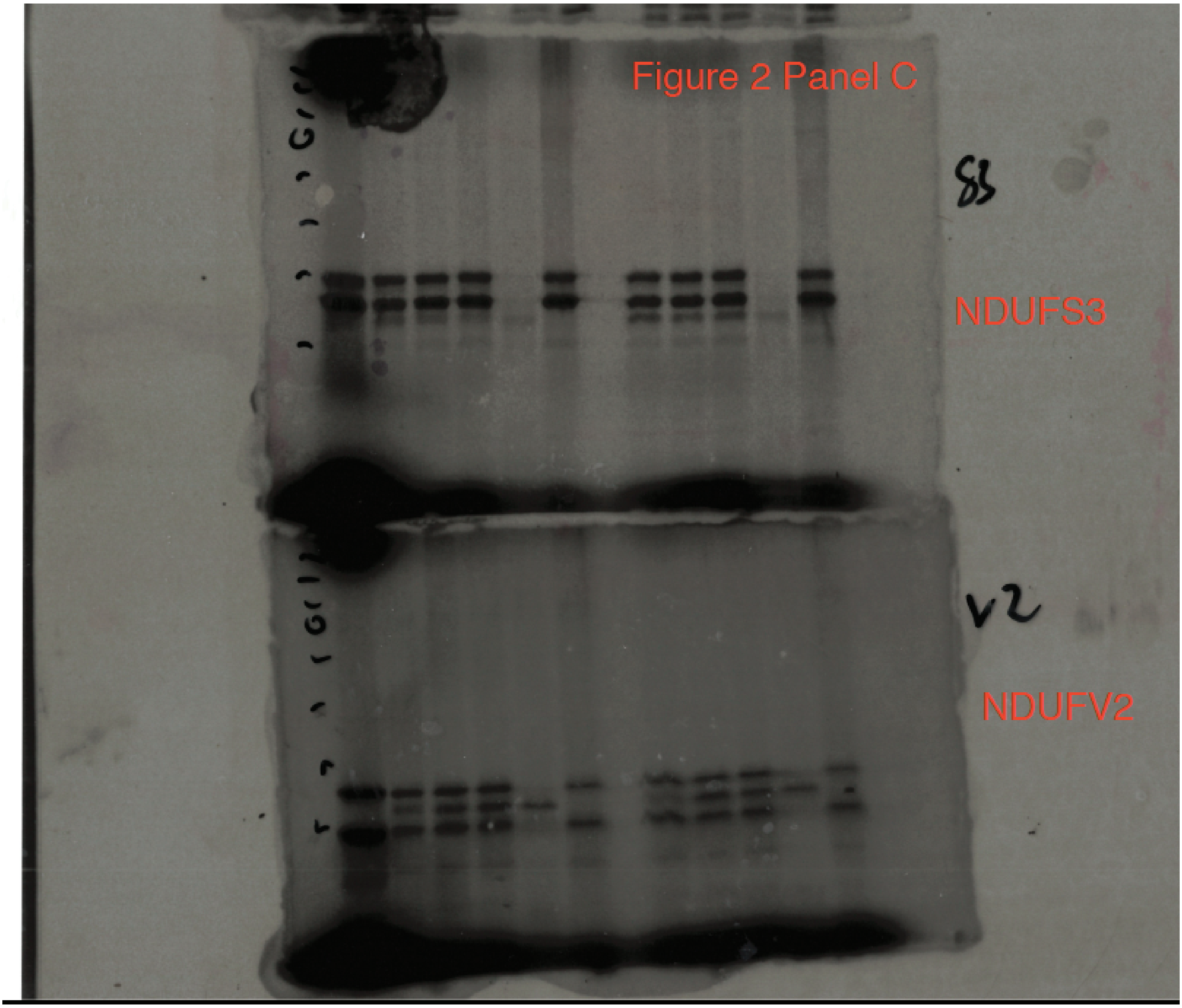

Figure 2 Panel D

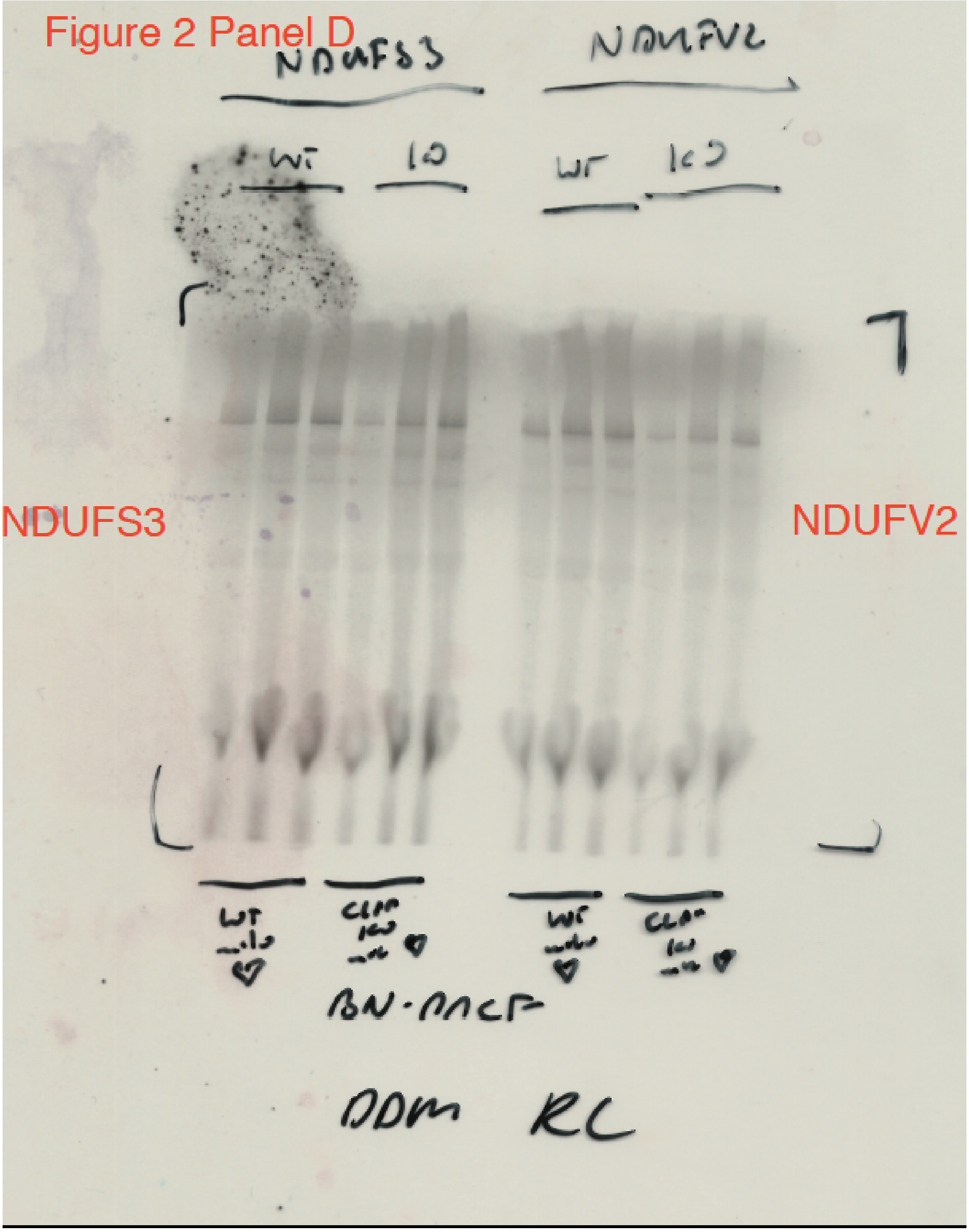

Figure 2 Panel E

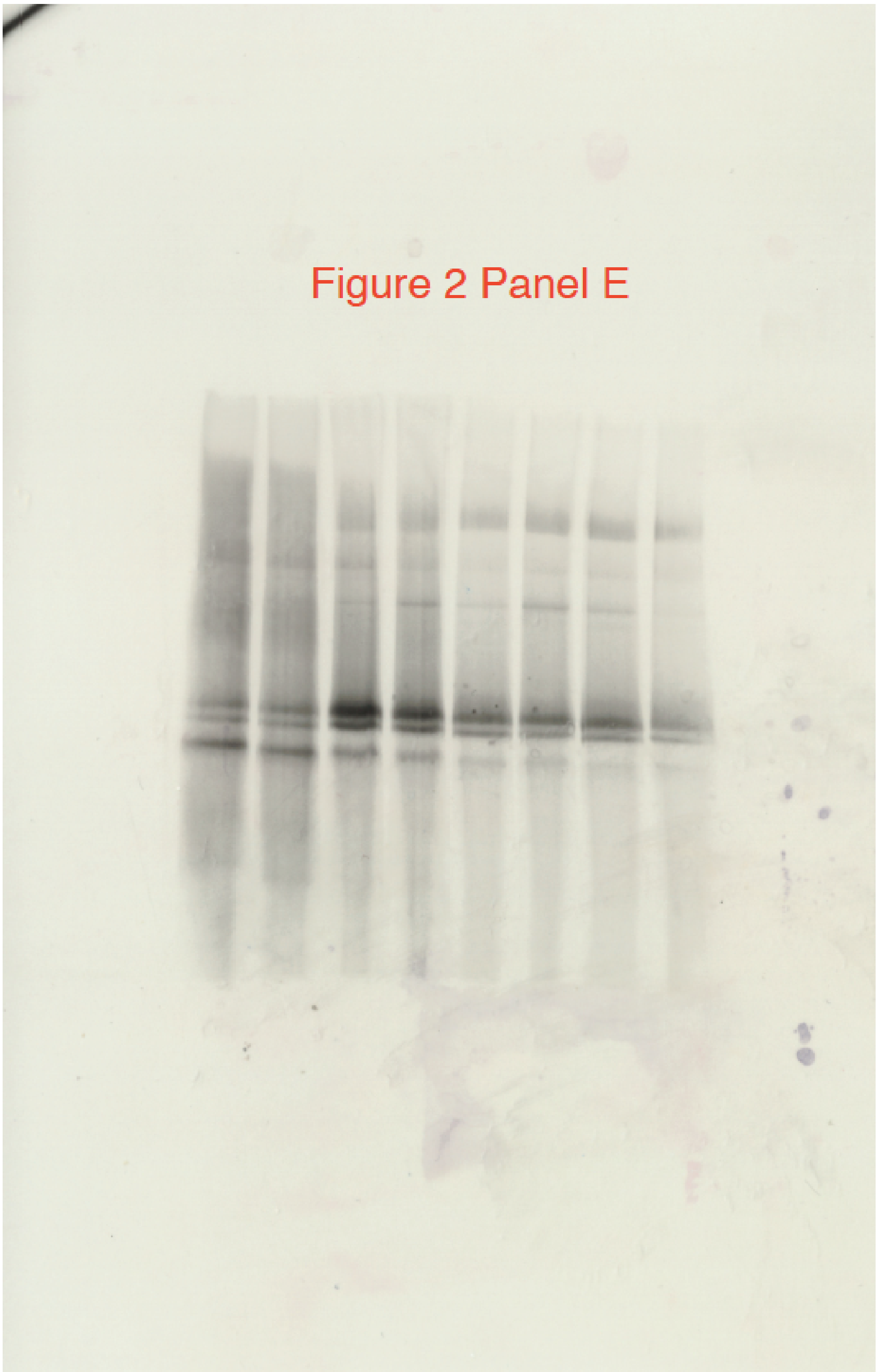

Figure 3 Panel A

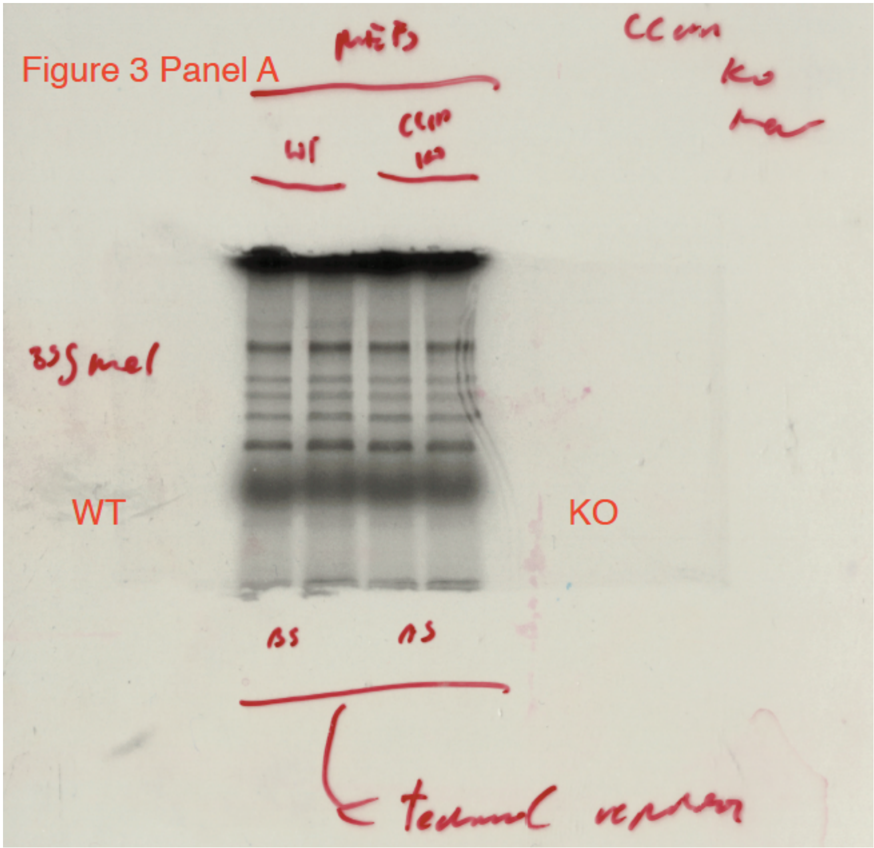

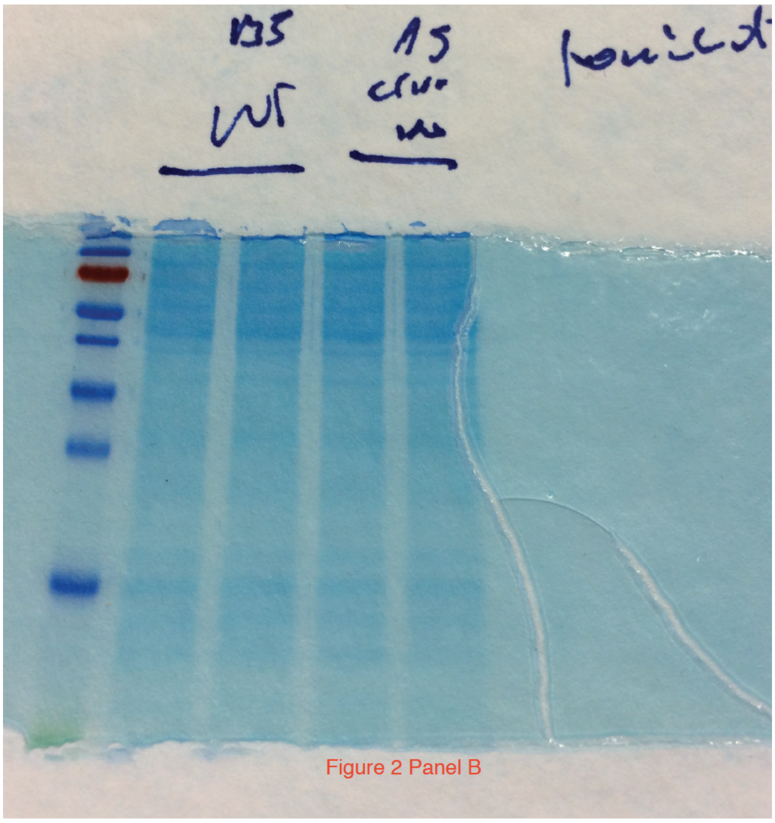

Figure 4 Panel A

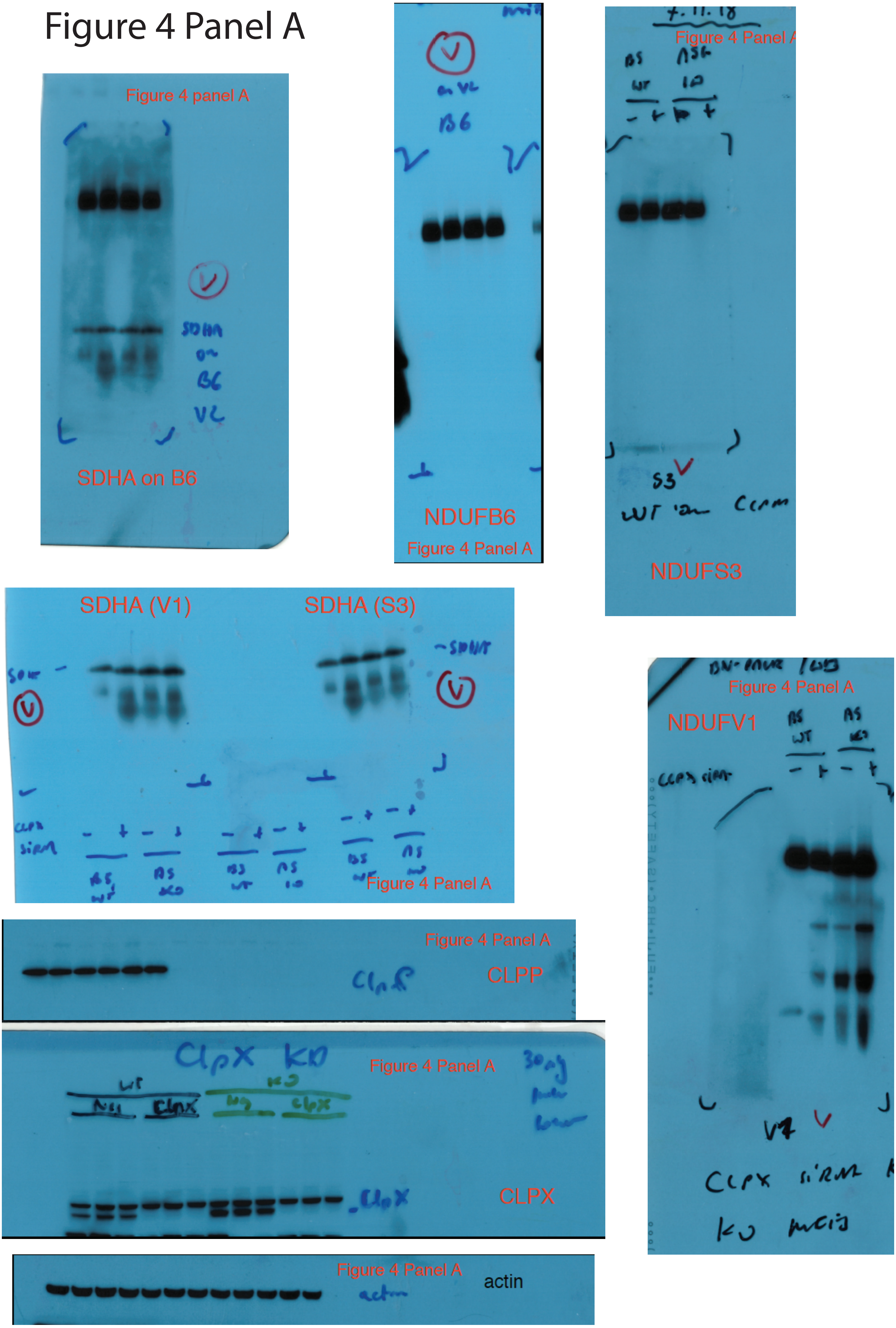

Figure 4 Panel B

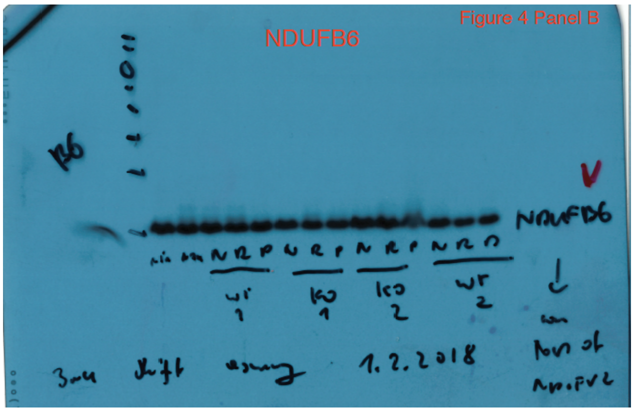

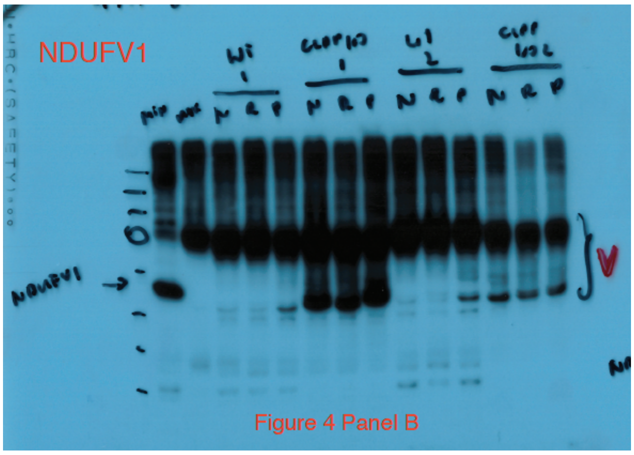

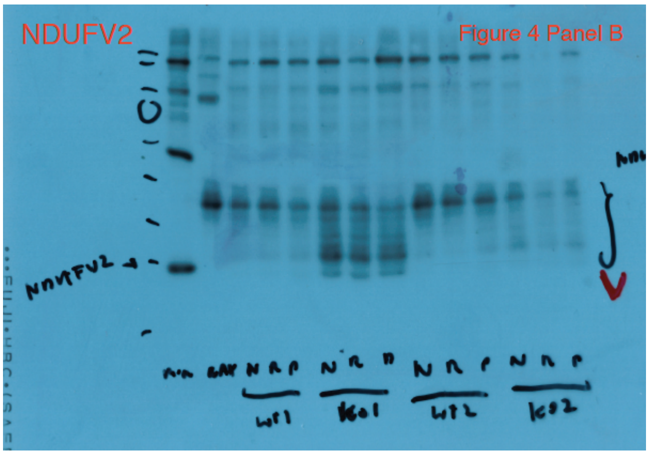

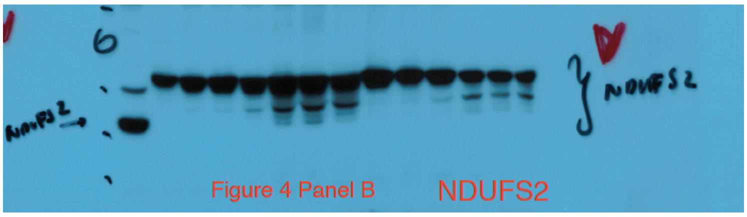

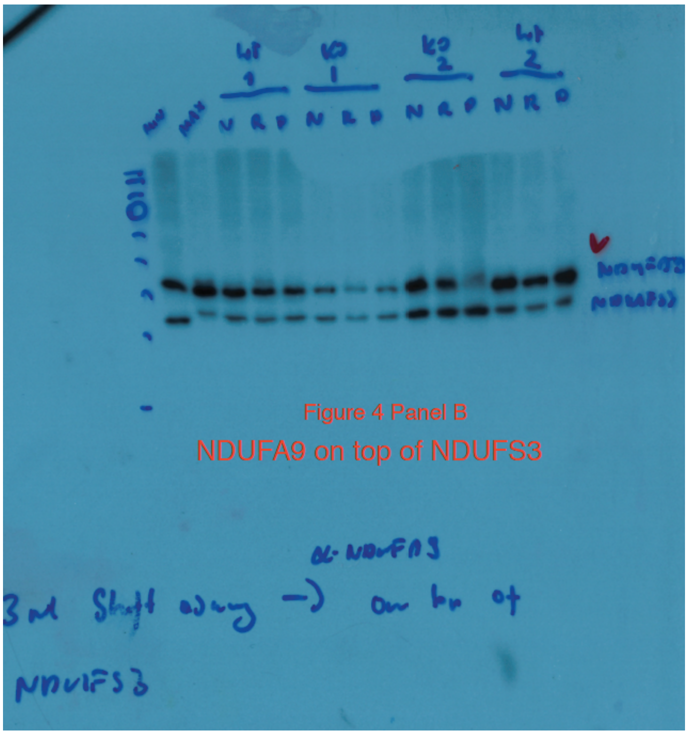

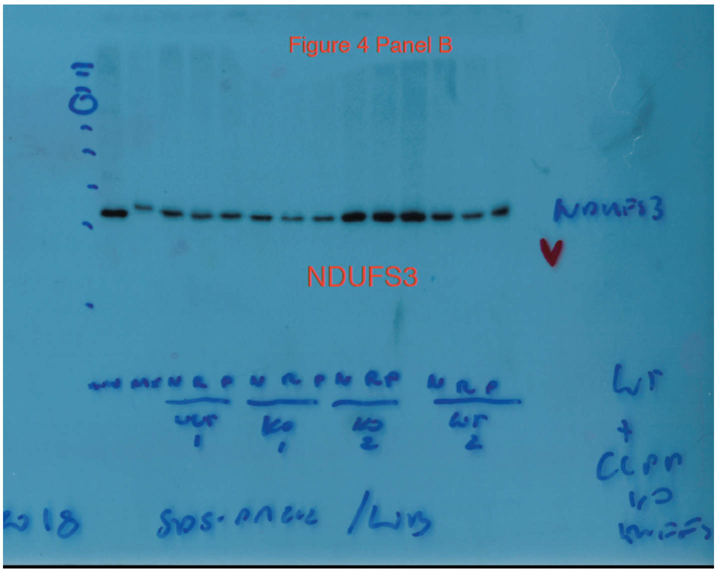

Figure 4 Panel C

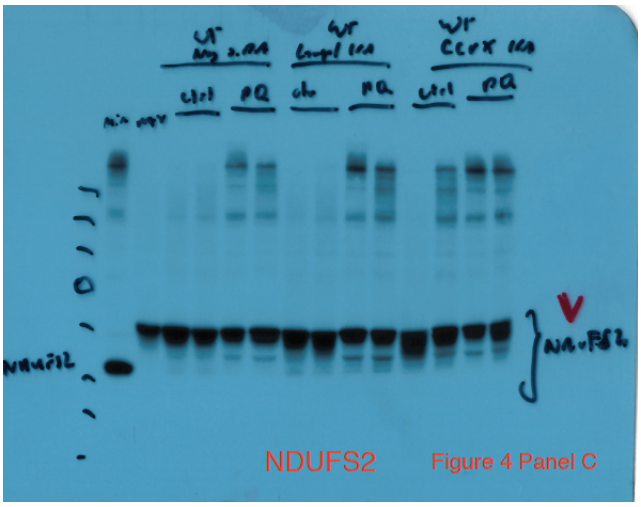

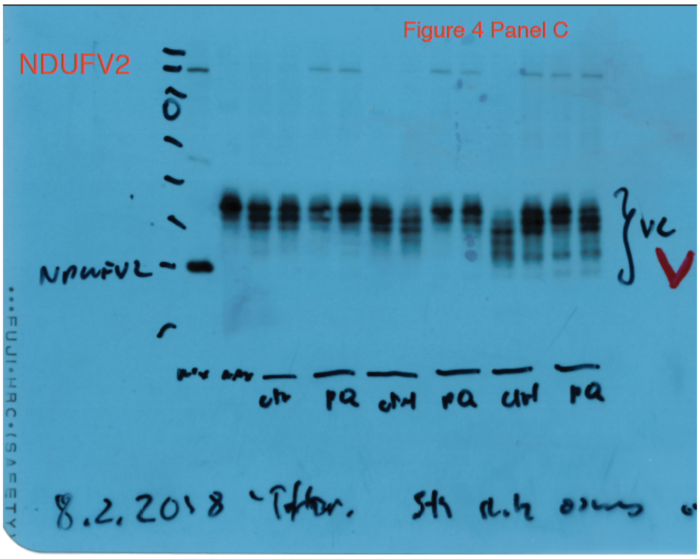

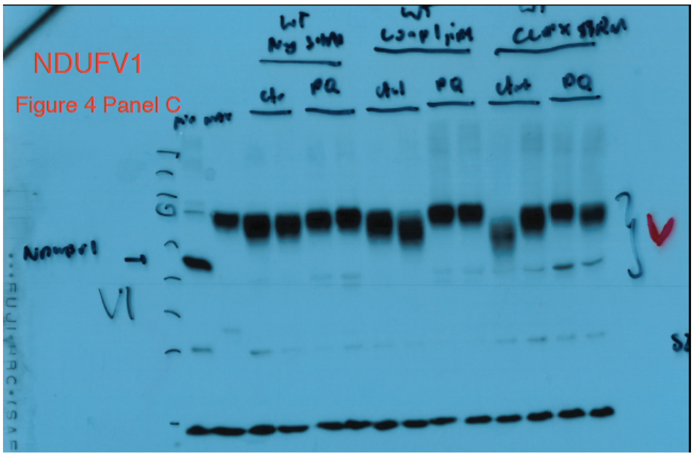

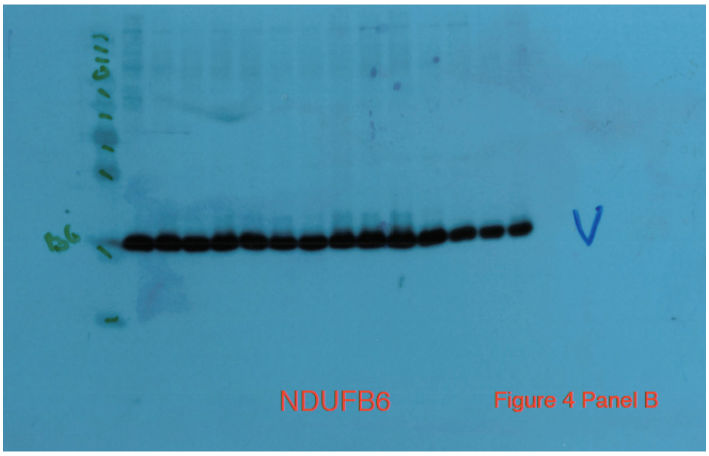

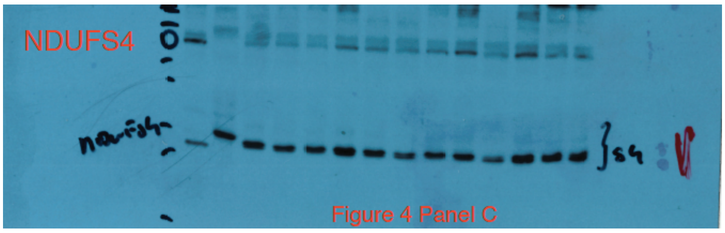

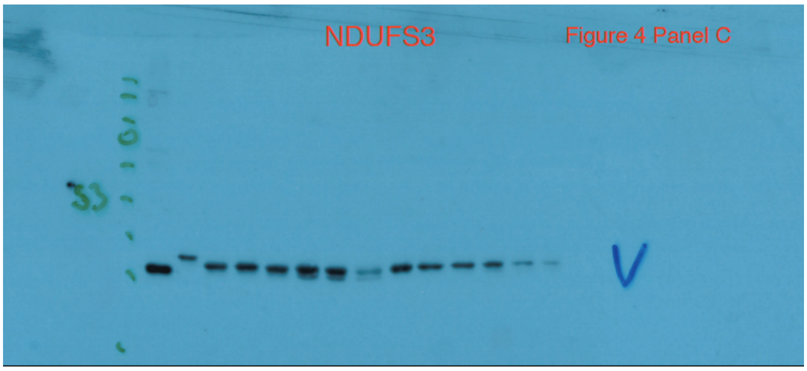

Figure 4 Panel D

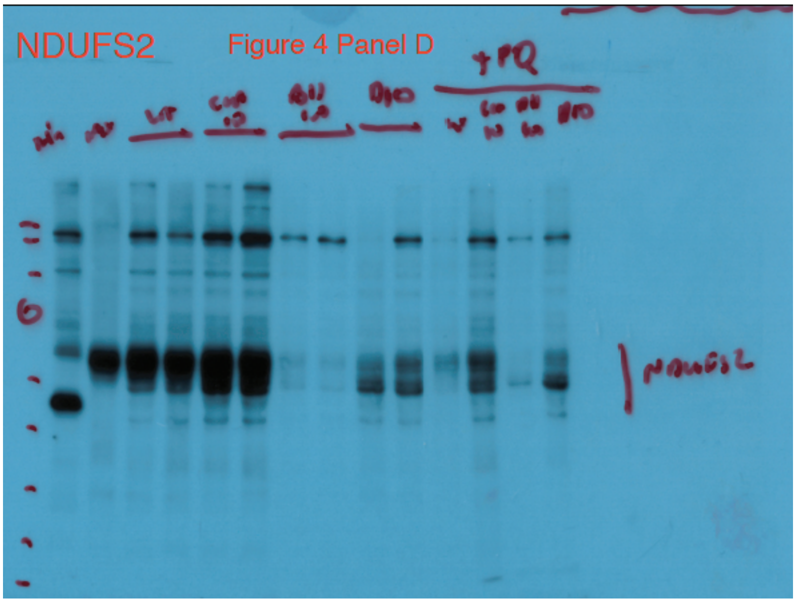

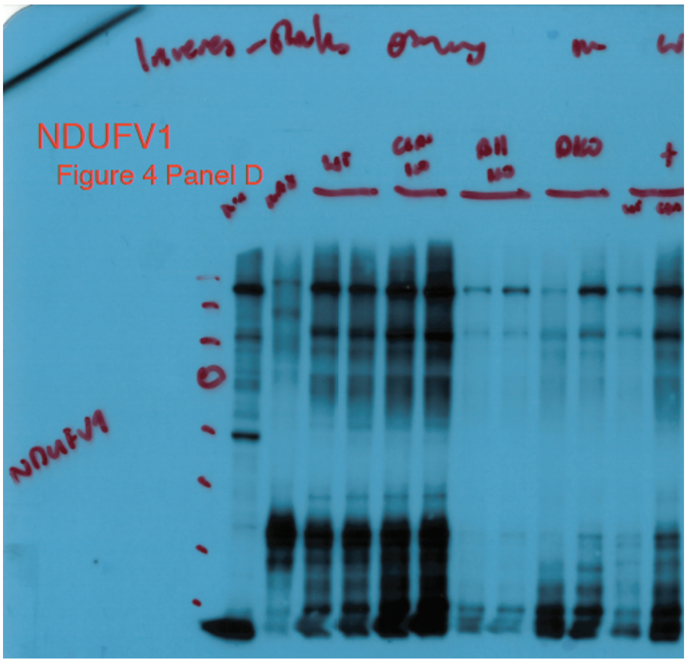

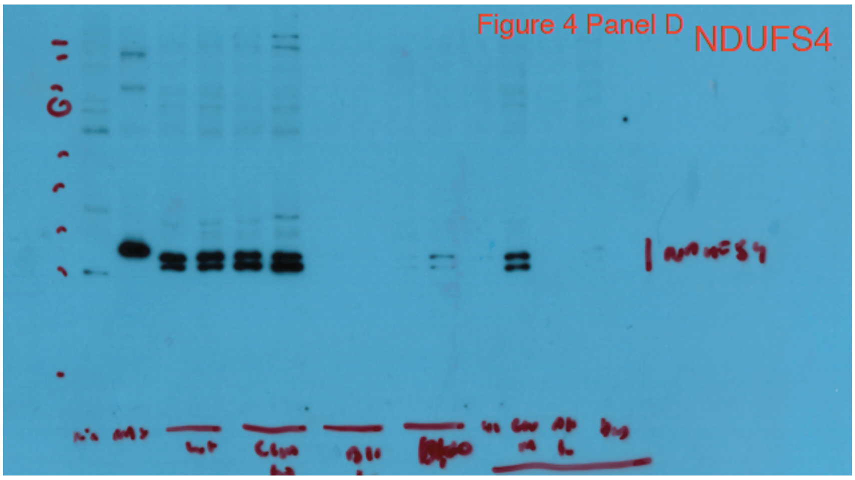

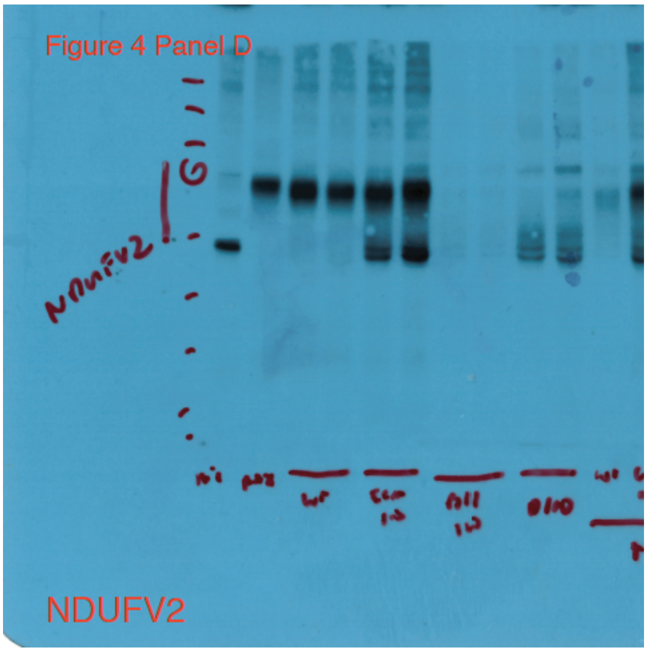

Figure 4 Panel E

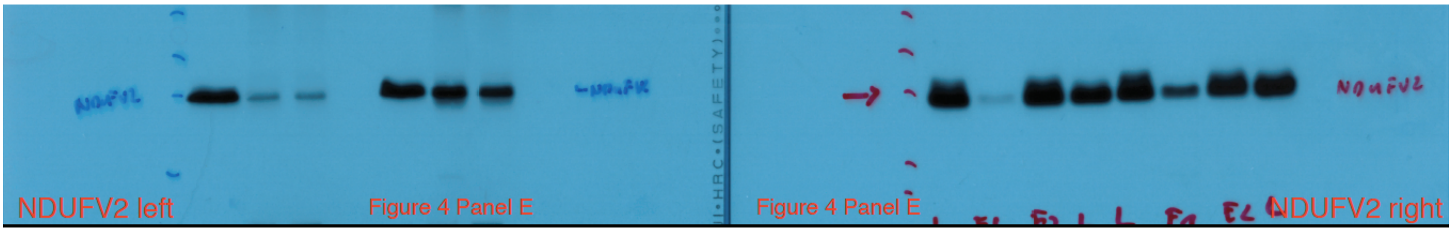

Figure 5 Panel A

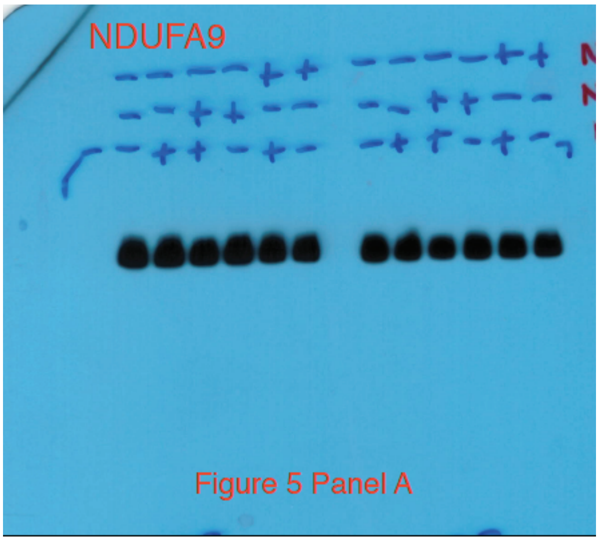

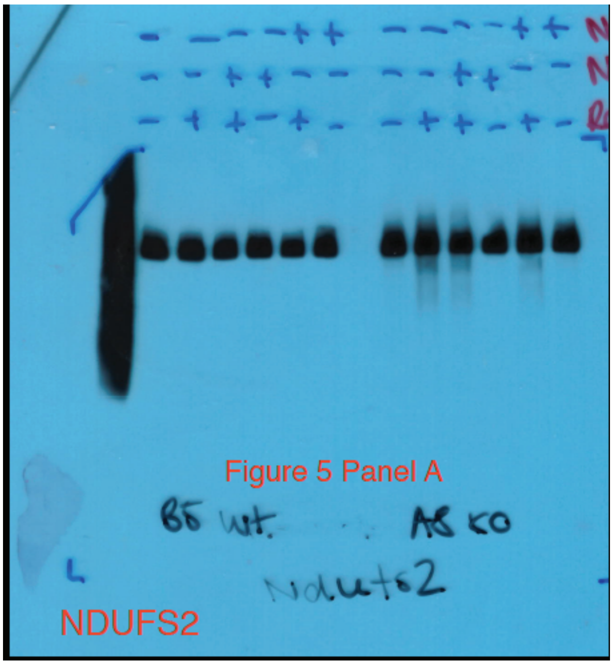

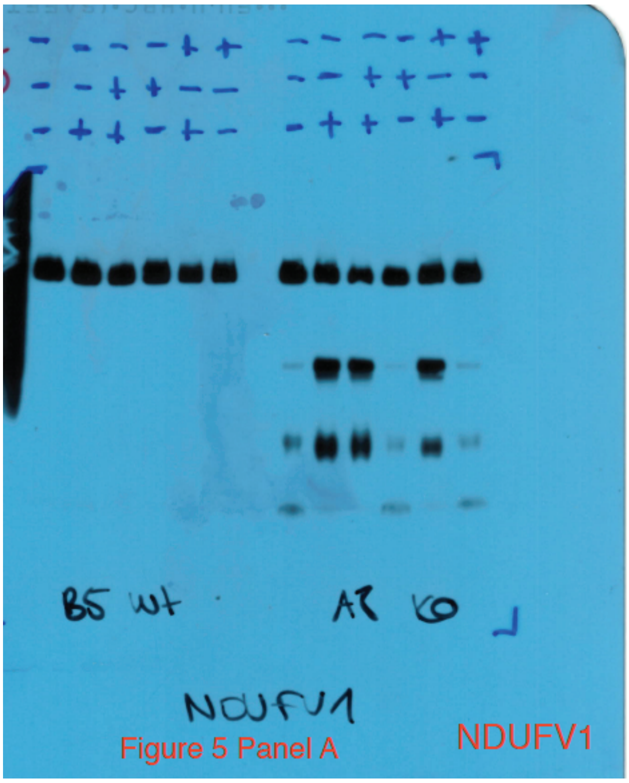

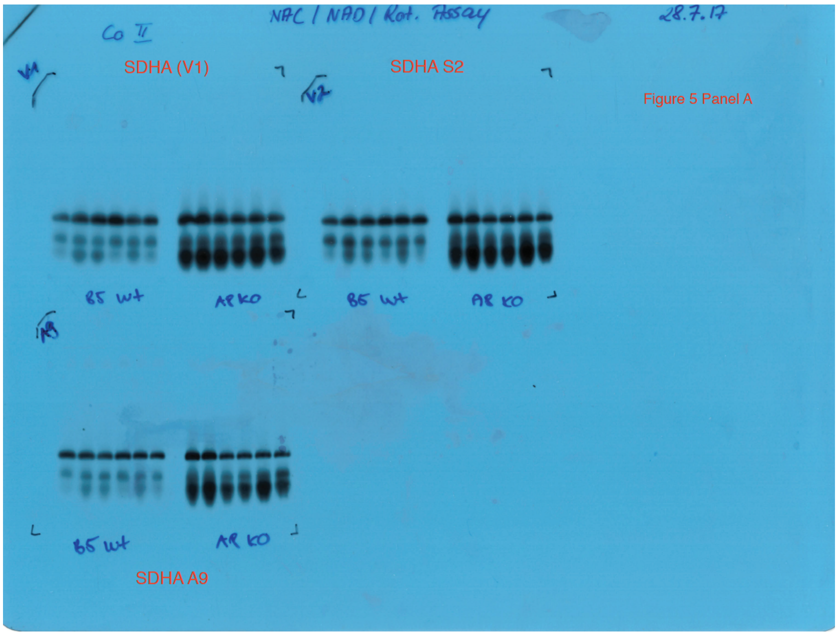

Figure 5 Panel B

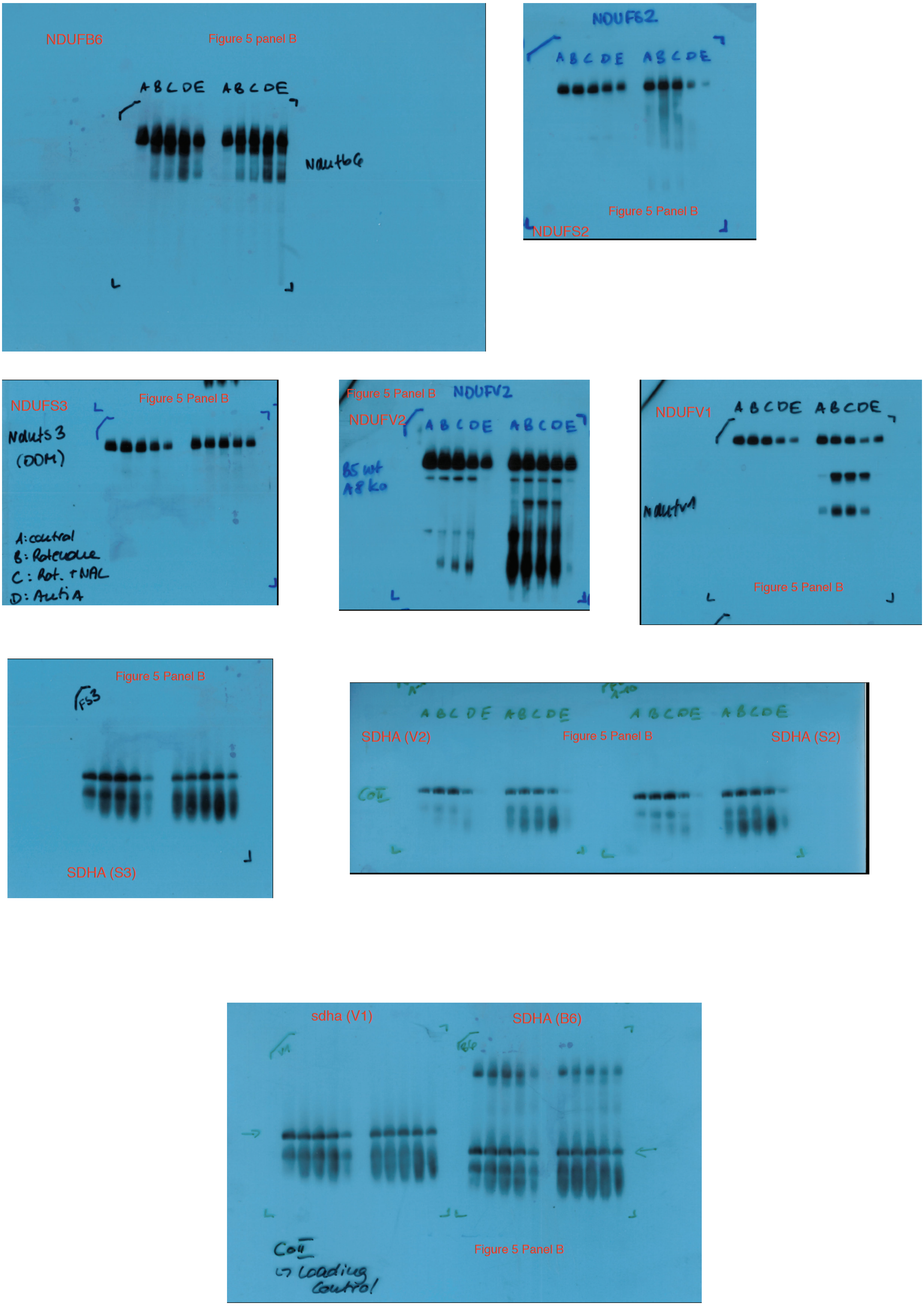

Figure 5 Panel D

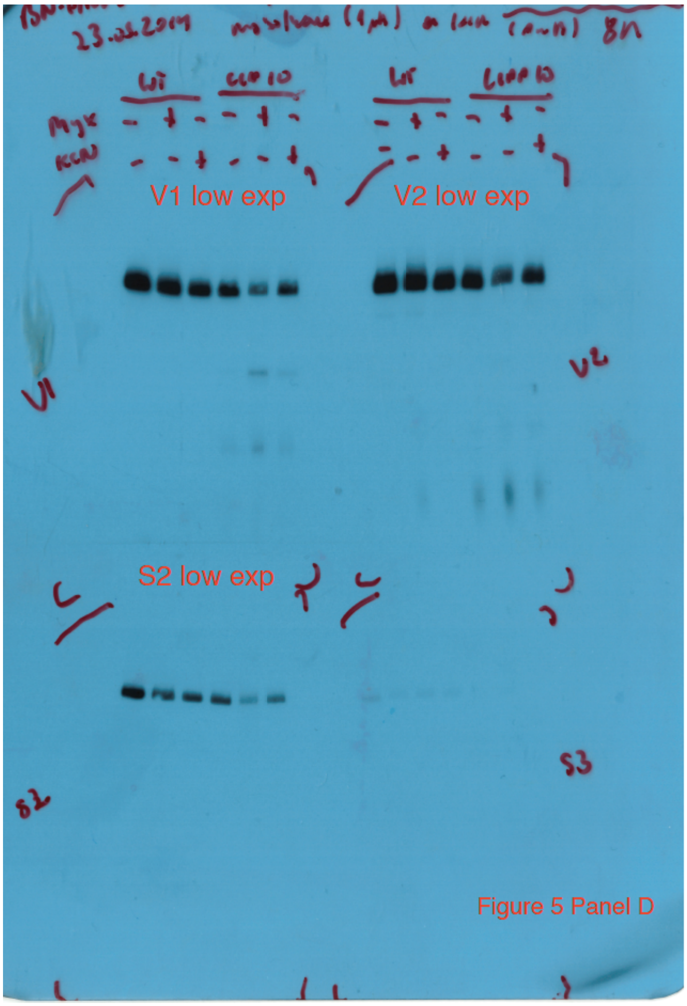

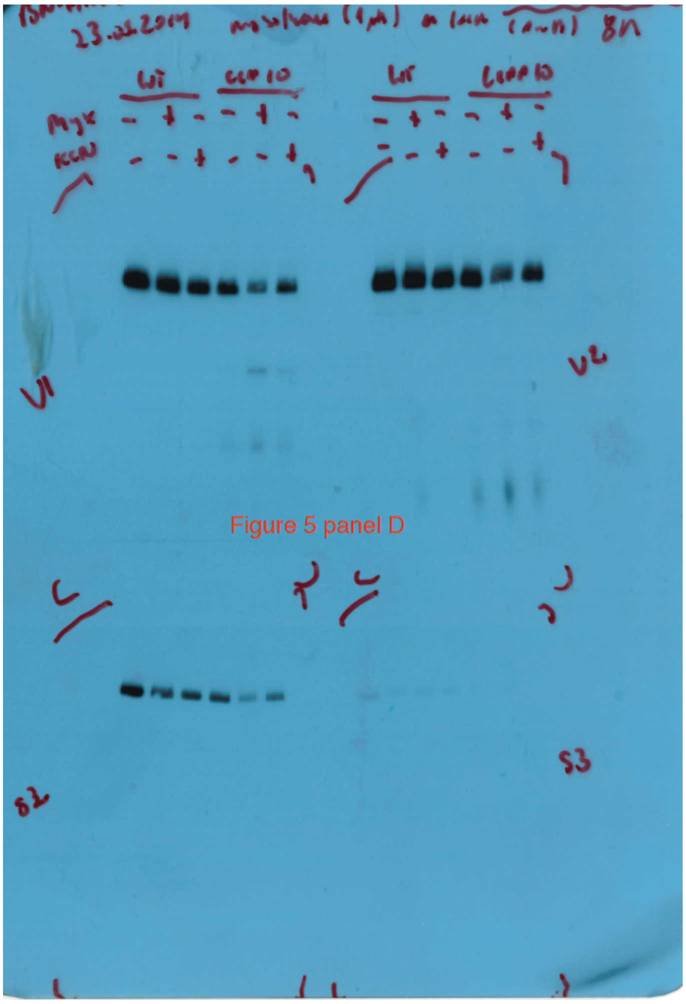

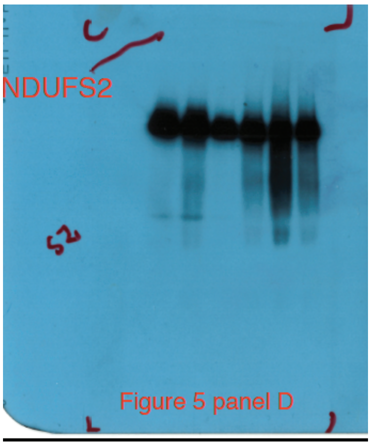

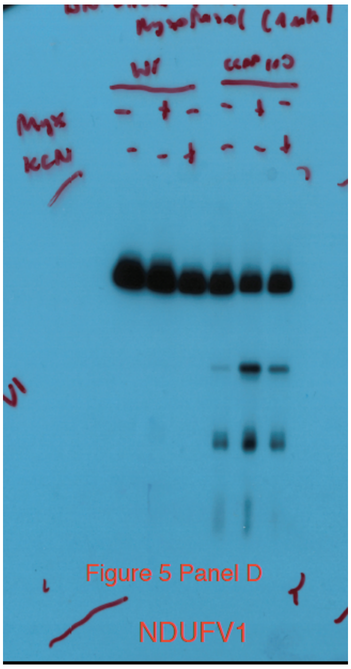

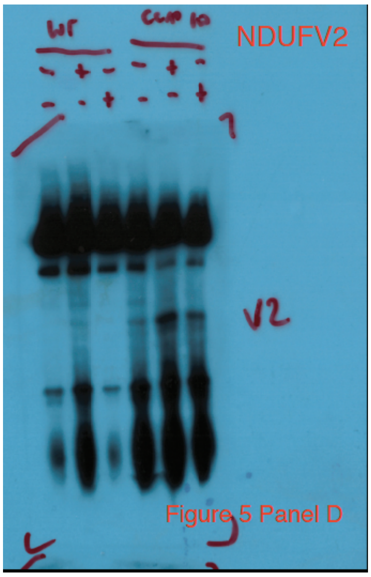

Figure 6 Panel A

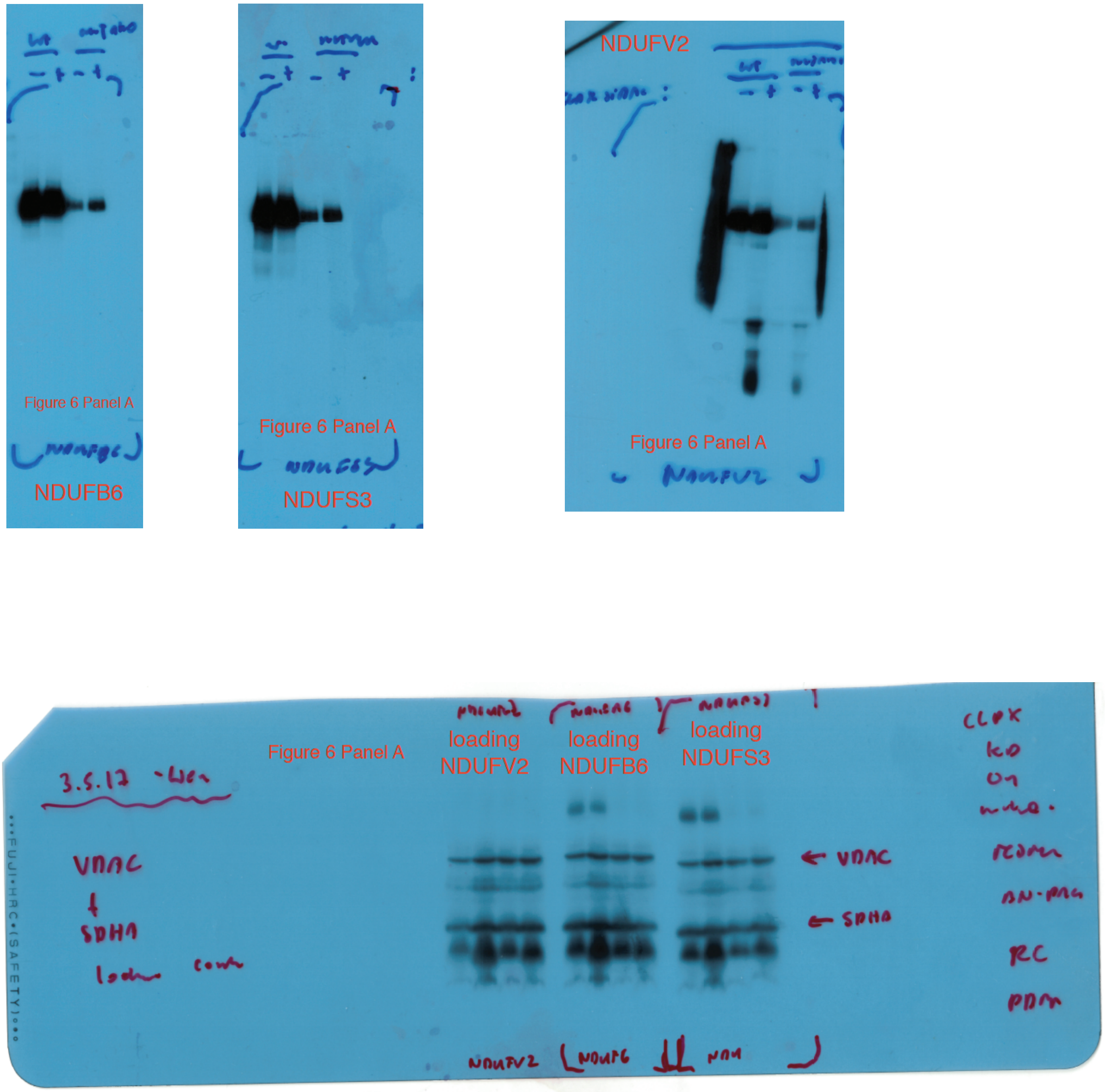

Figure 6 Panel C

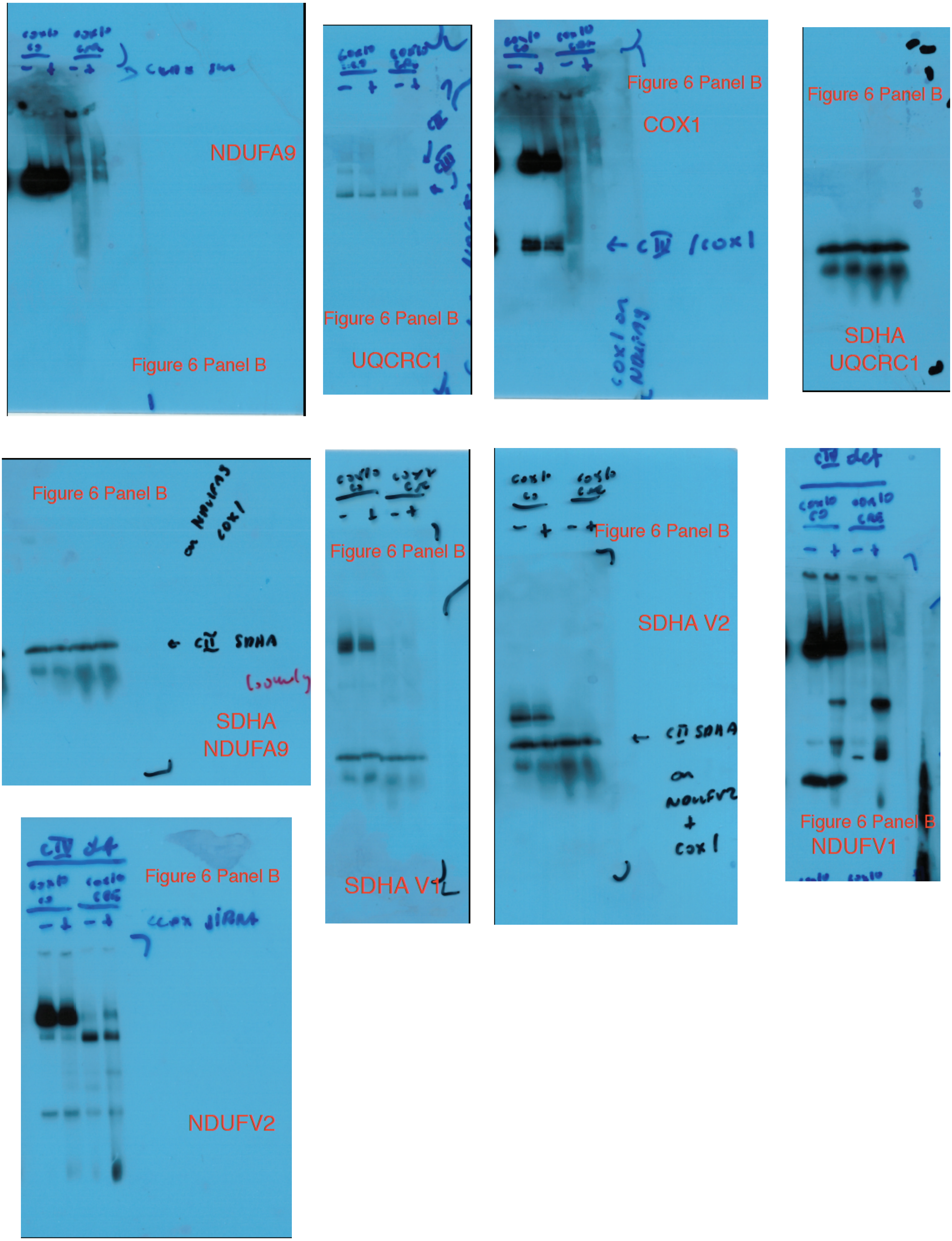

Figure 6 Panel E

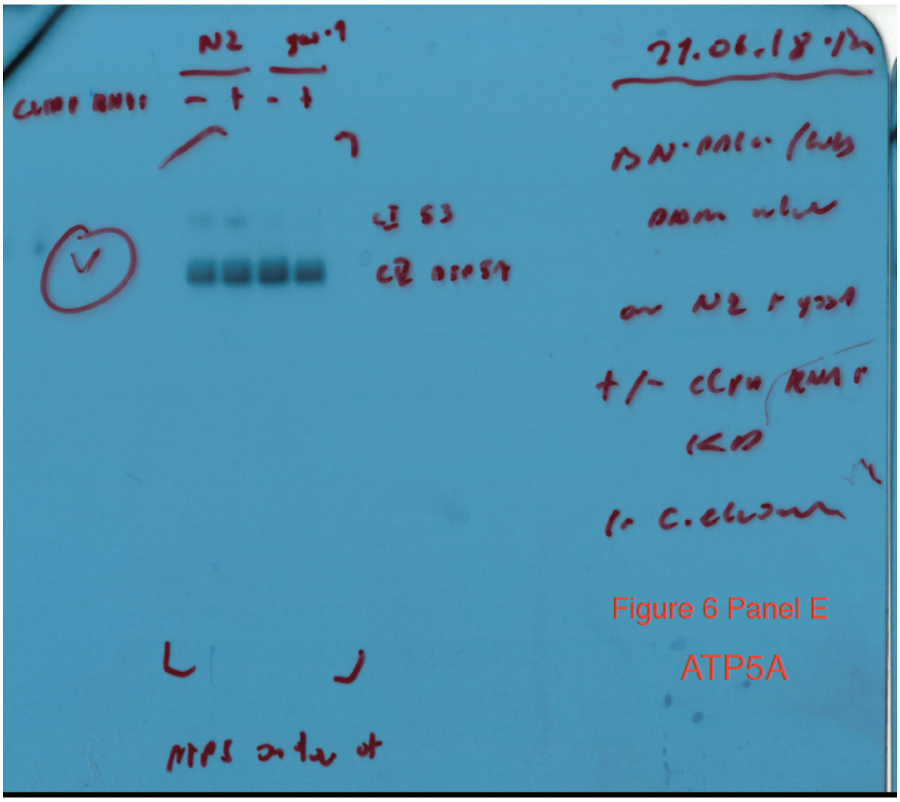

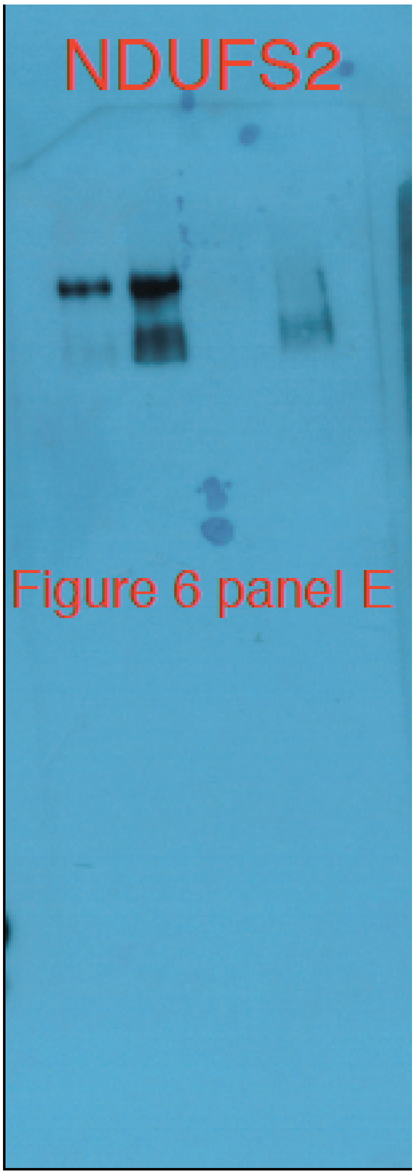

Supplementary Figure 1 Panel D

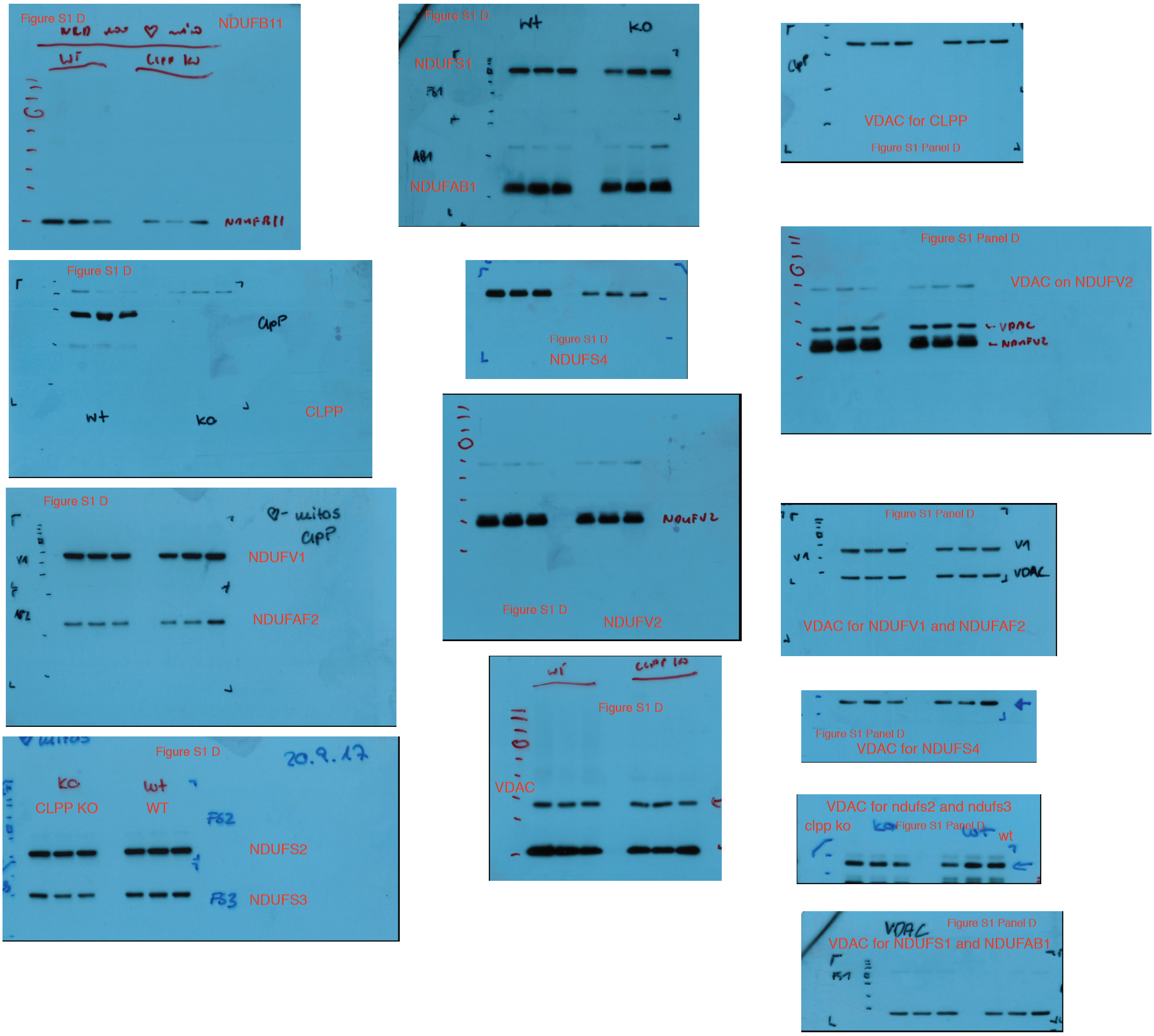

Supplementary Figure 2 Panel A

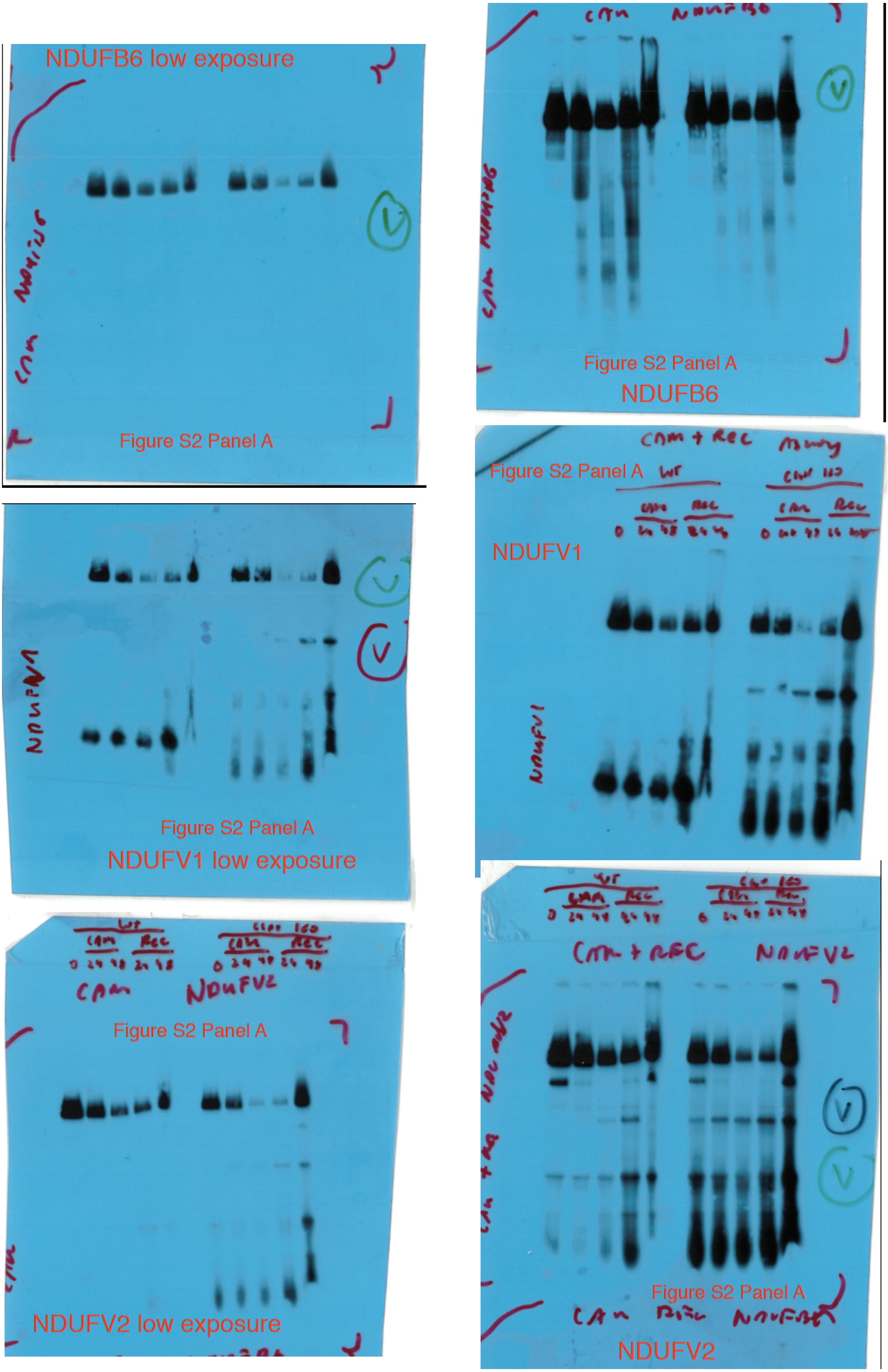

Supplementary Figure 2 Panel B

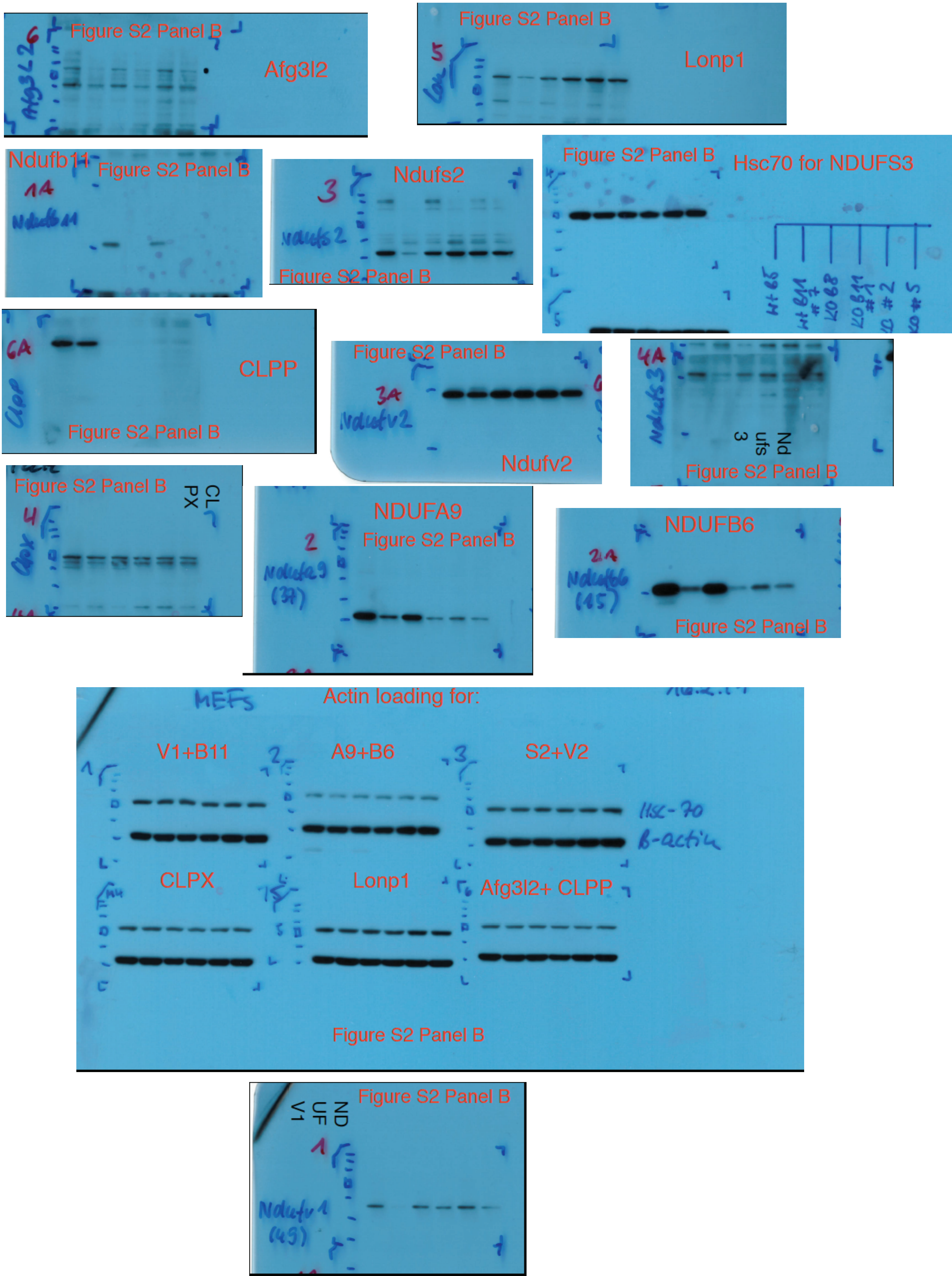

Supplementary Figure 3 Panel A

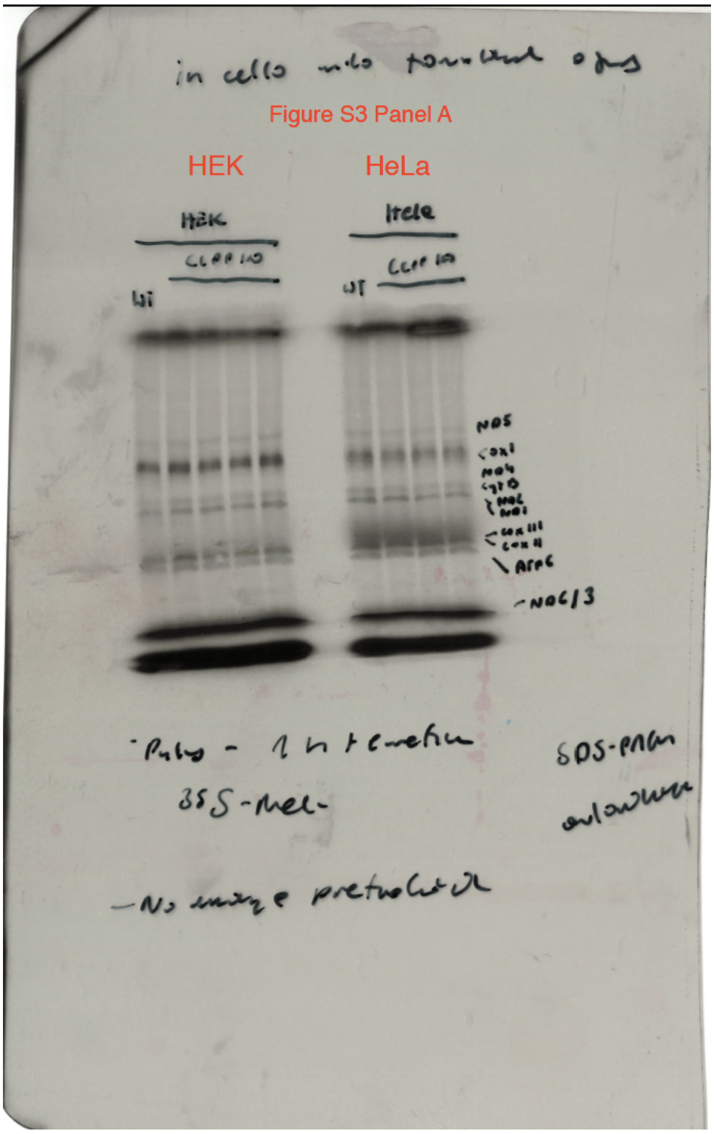

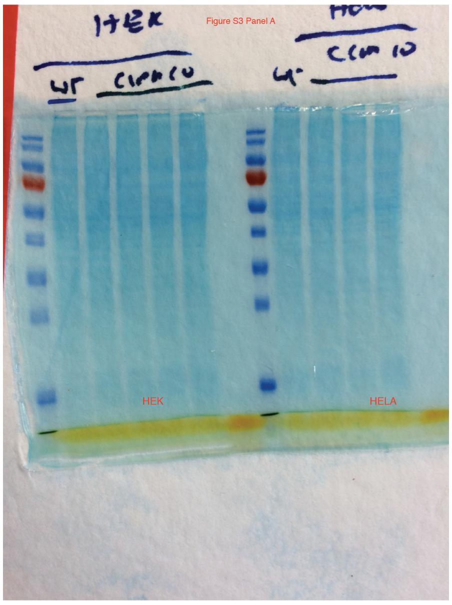

Supplementary Figure 3 Panel B

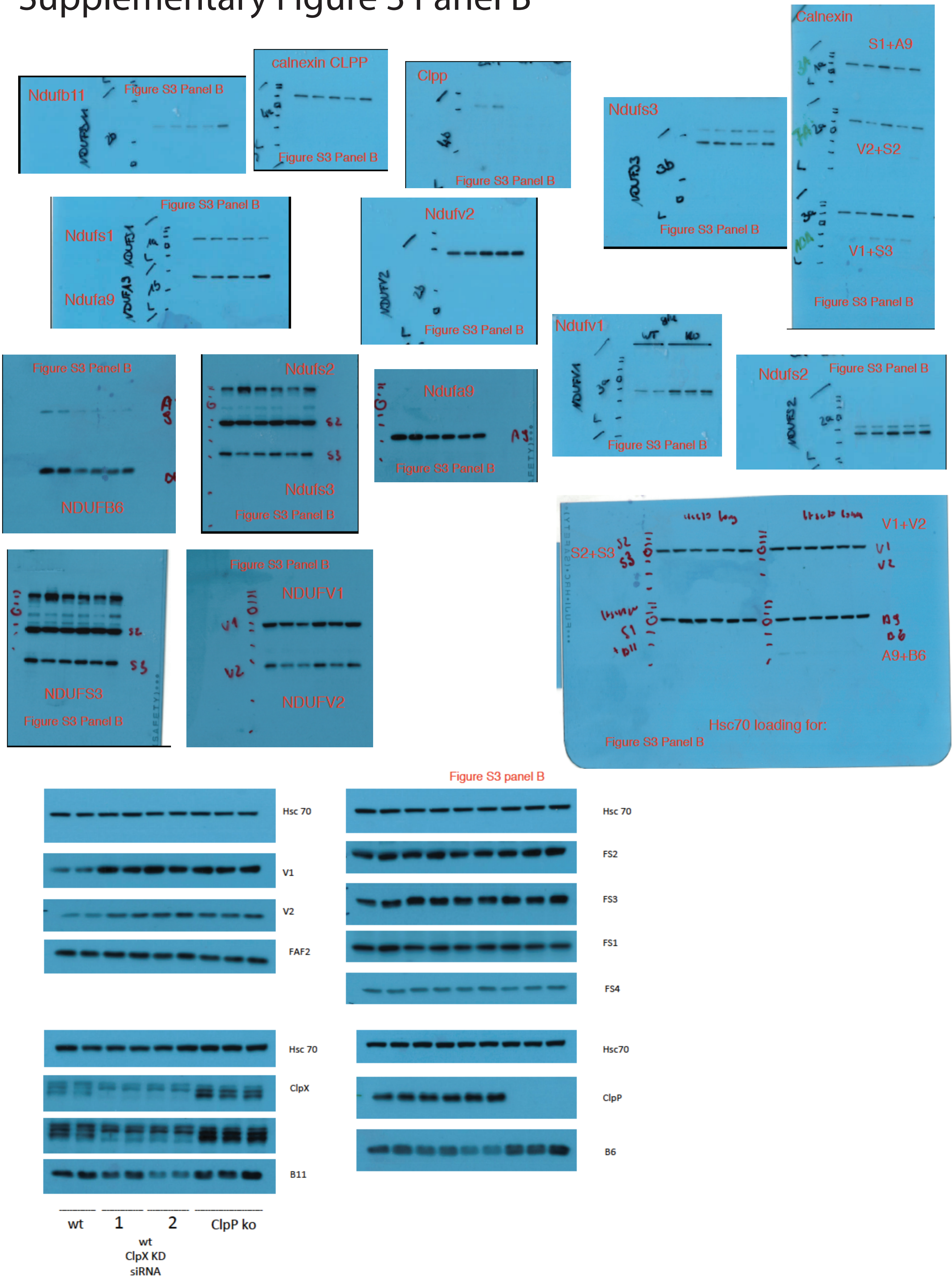

Supplementary Figure 4 Panel A

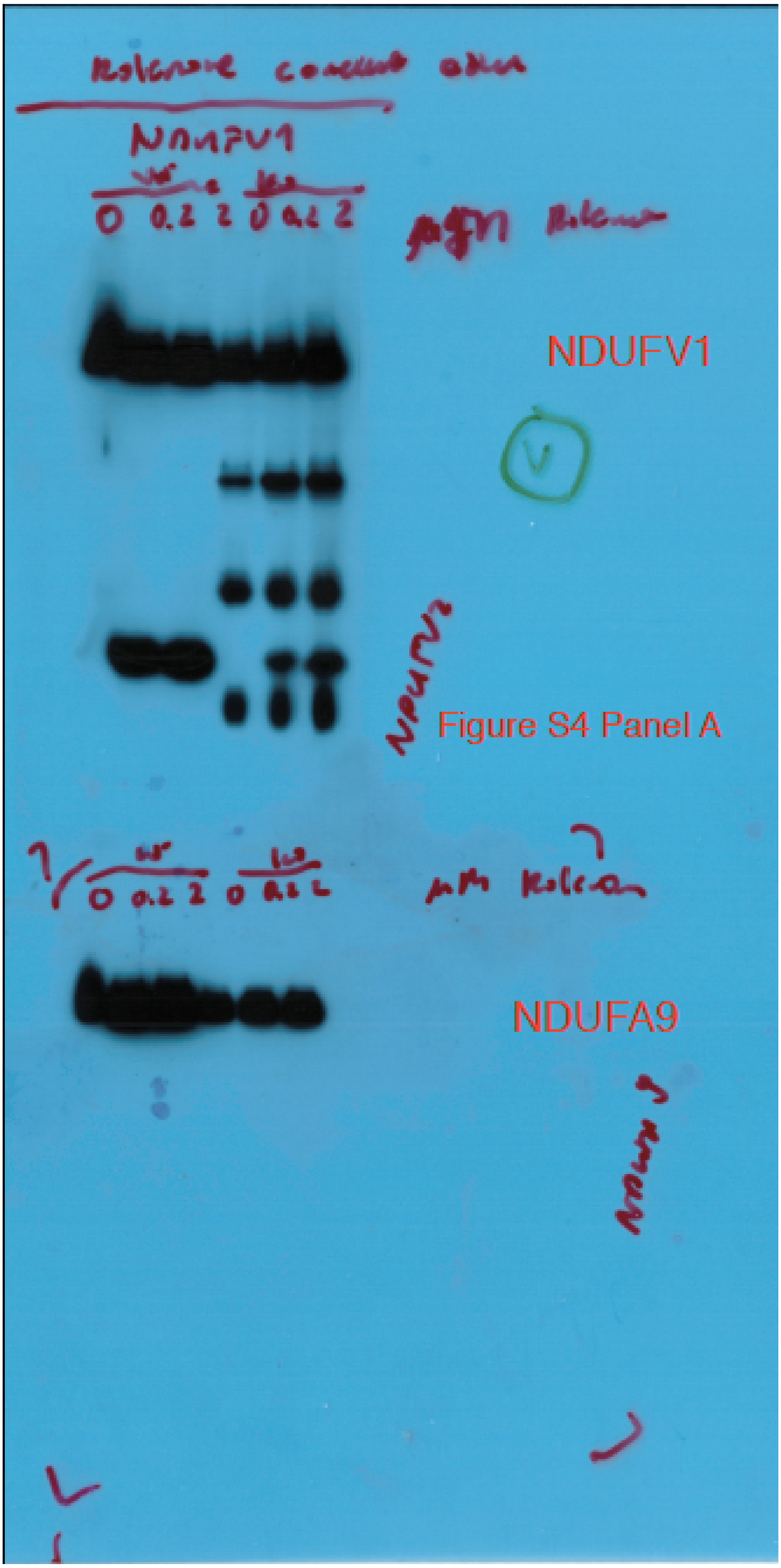

Supplementary Figure 4 Panel B

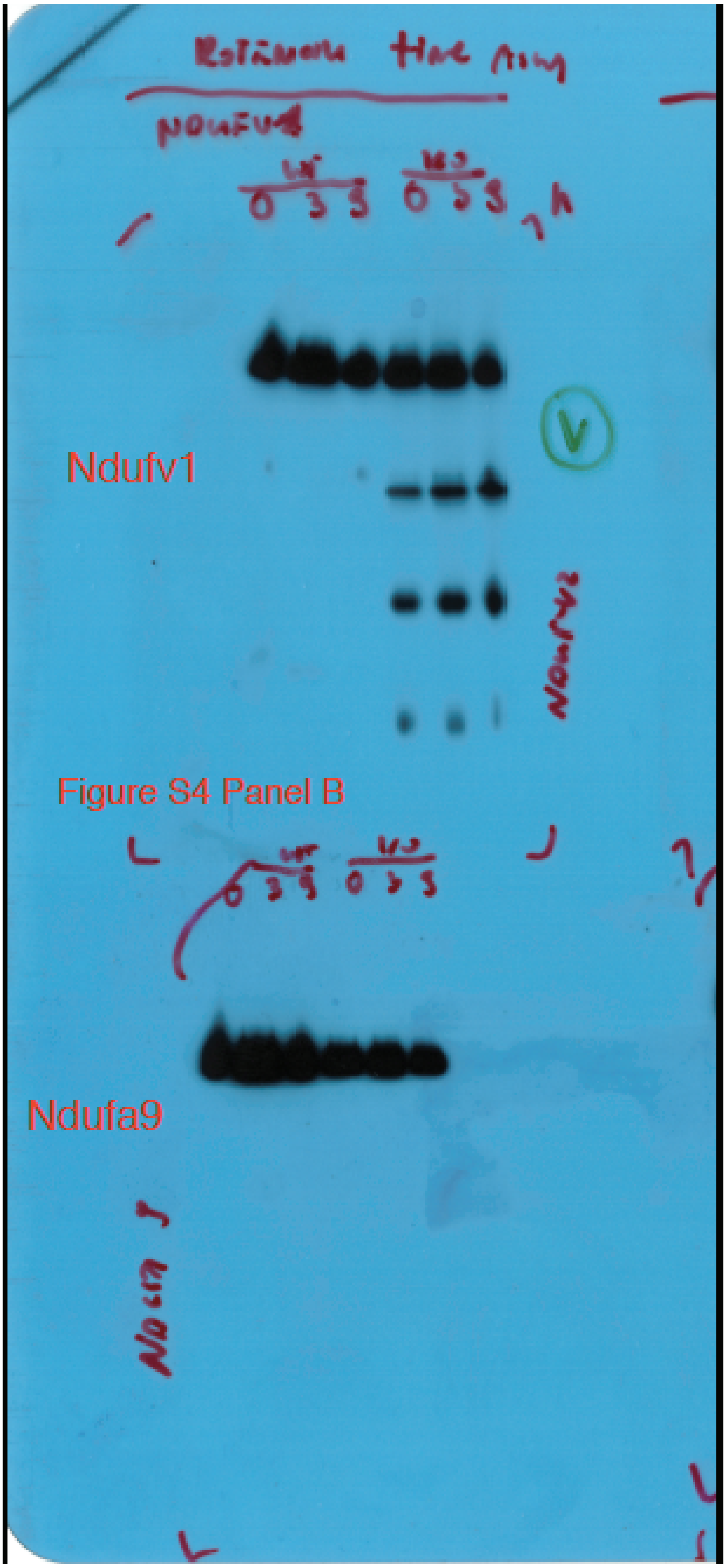

Supplementary Figure 4 Panel C

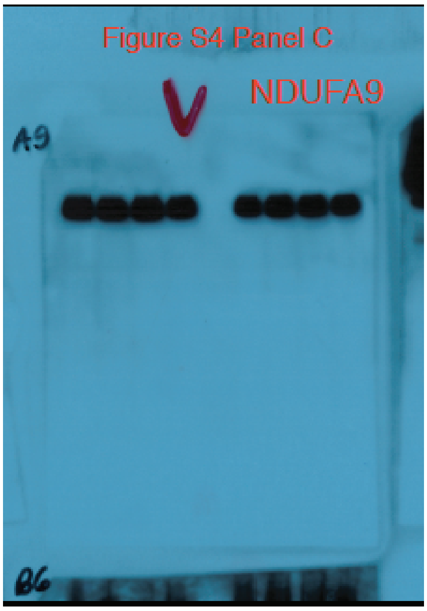

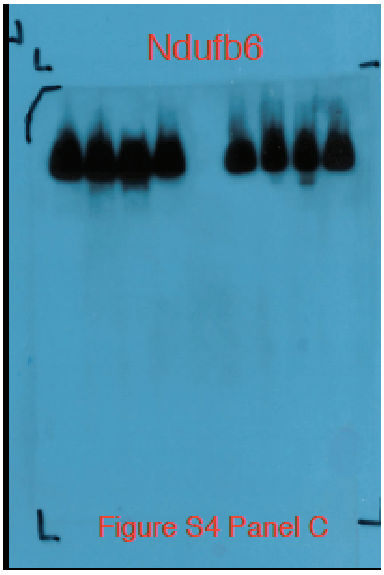

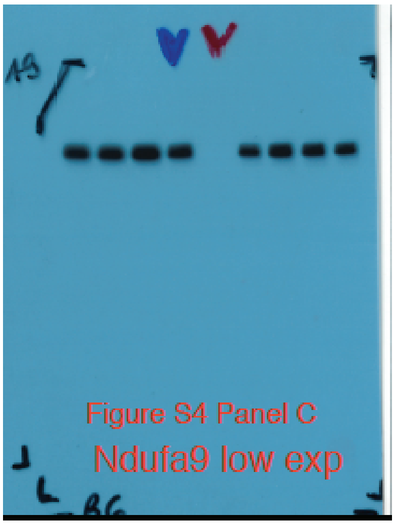

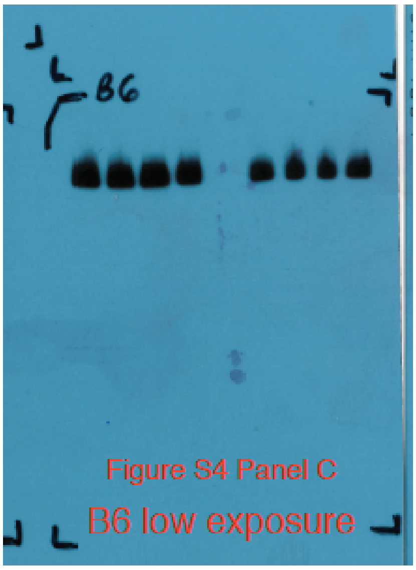

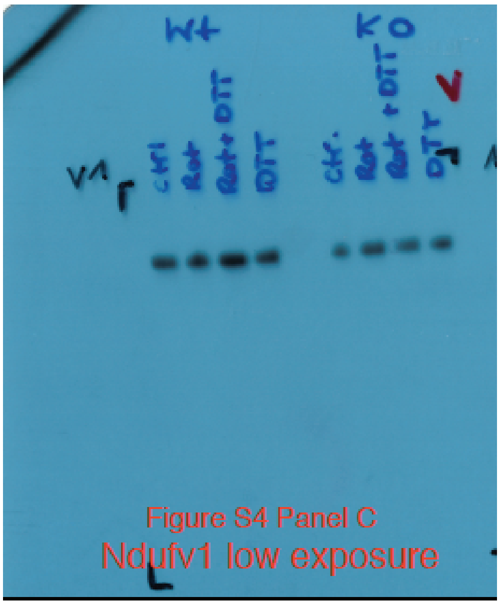

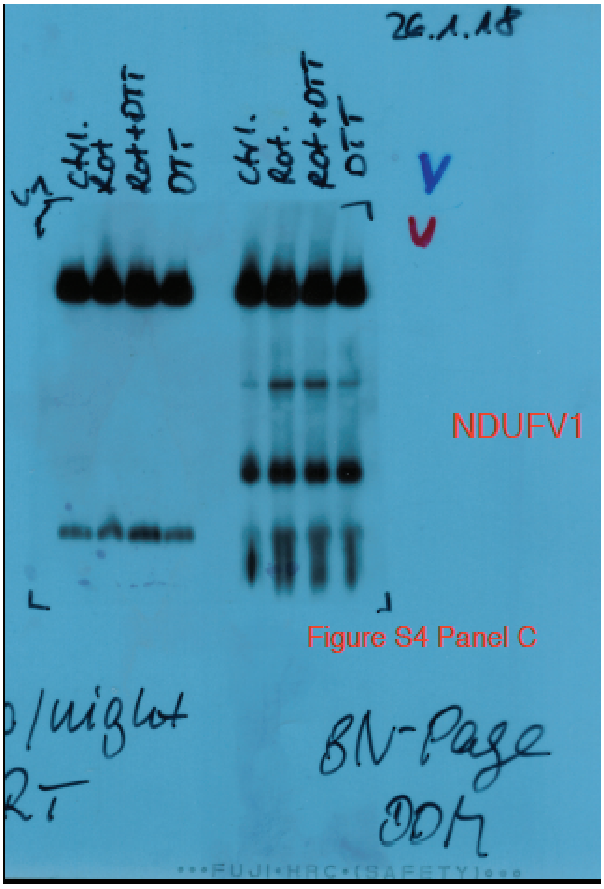

Supplementary Figure 4 Panel D

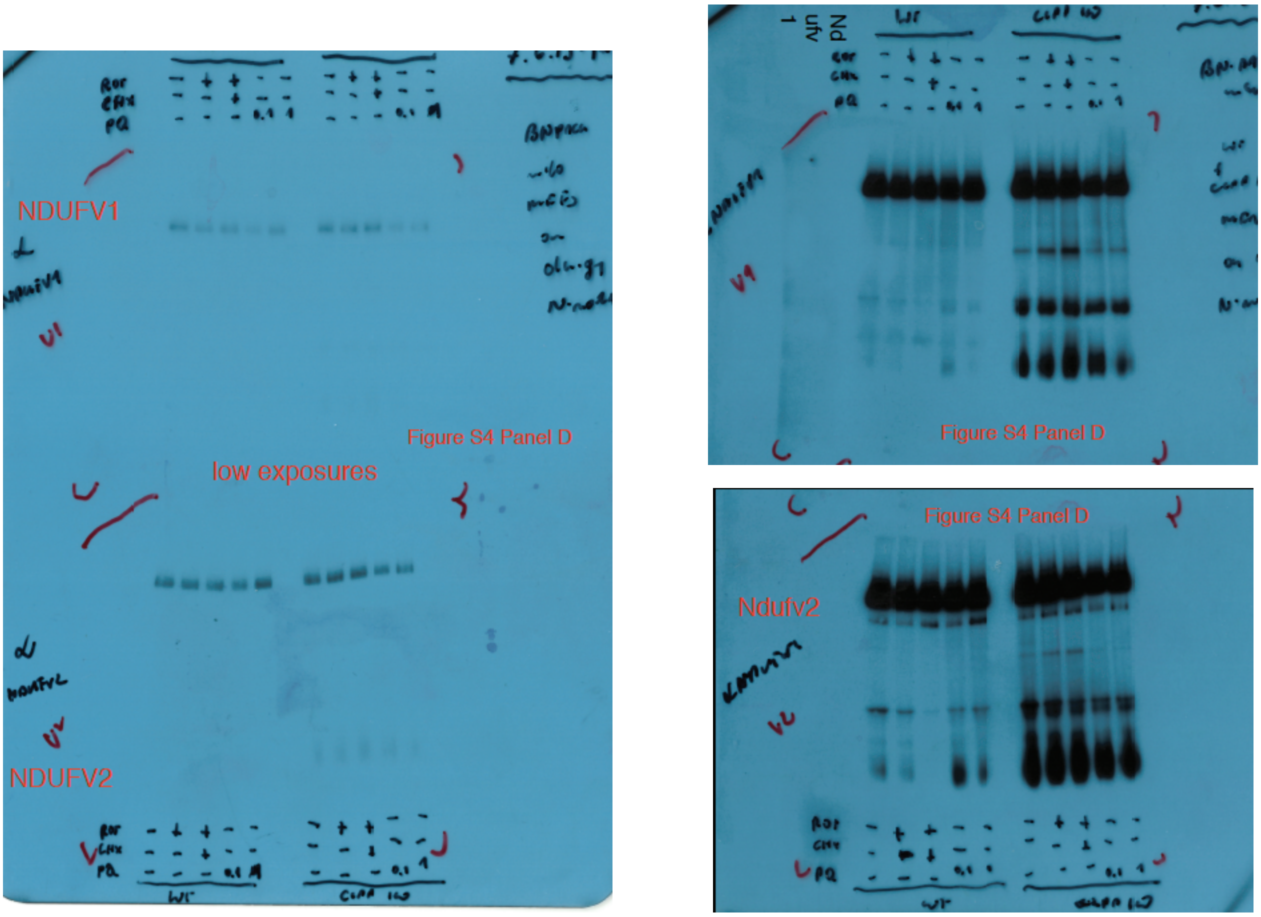

Supplementary Figure 4 Panel E

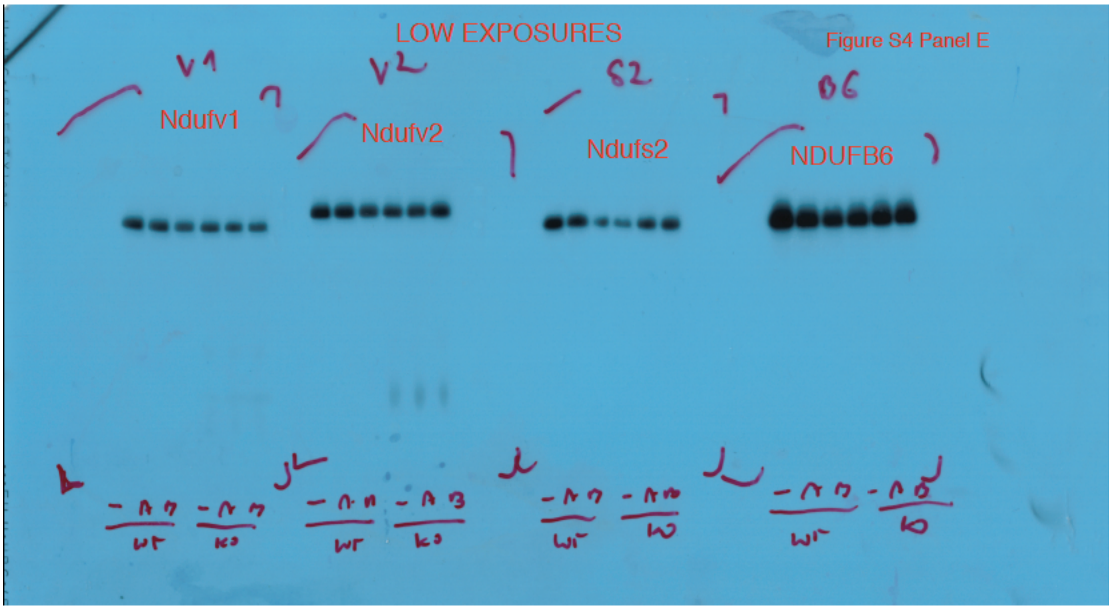

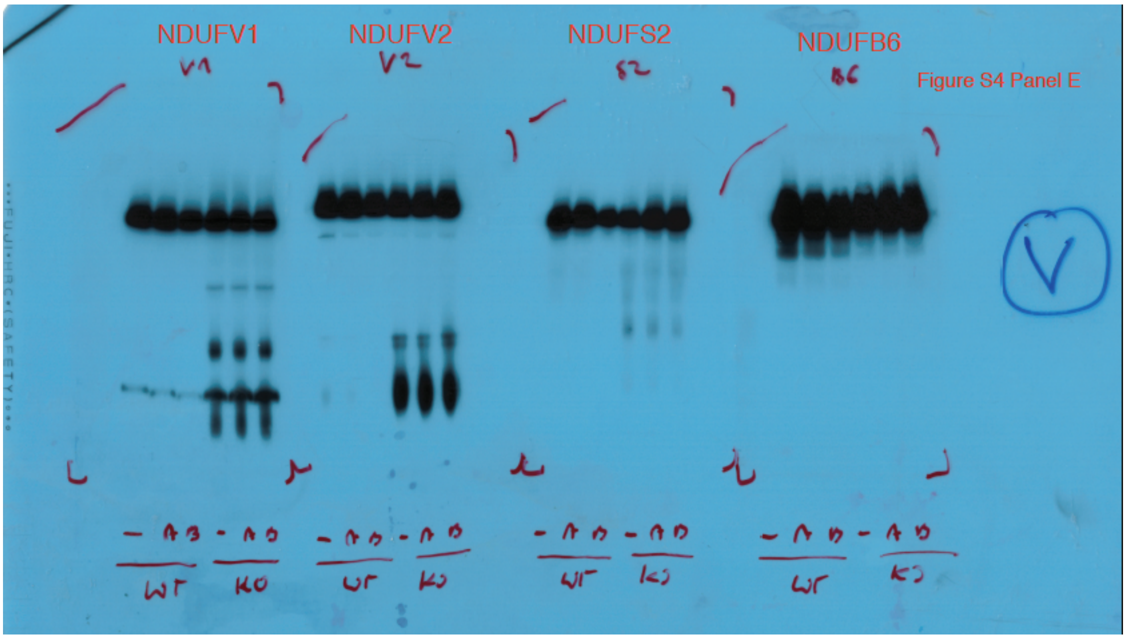

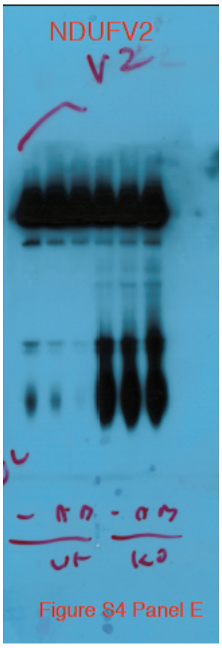

Supplementary Figure 4 Panel F

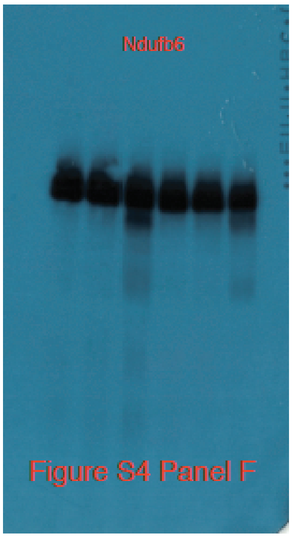

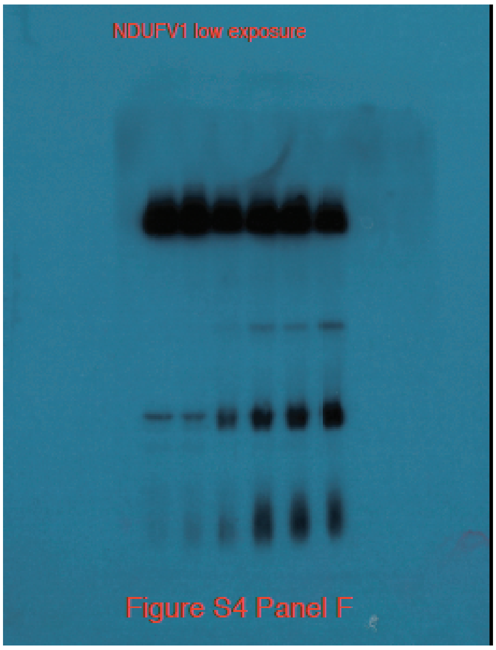

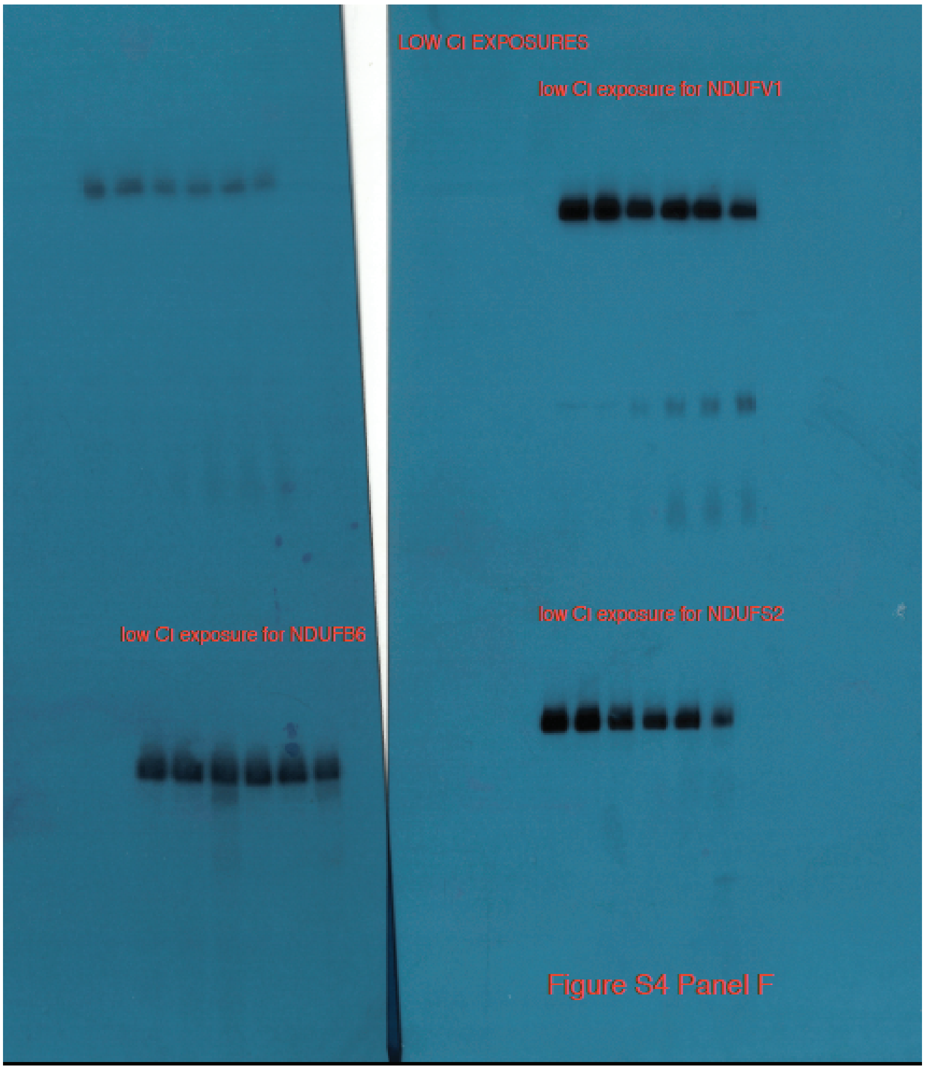

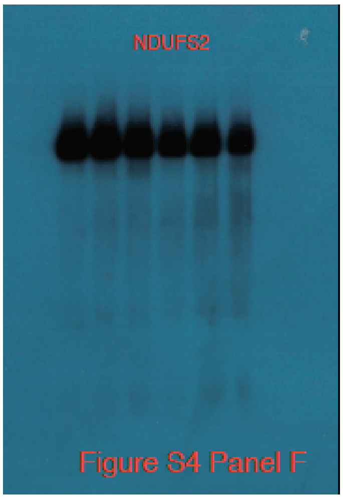

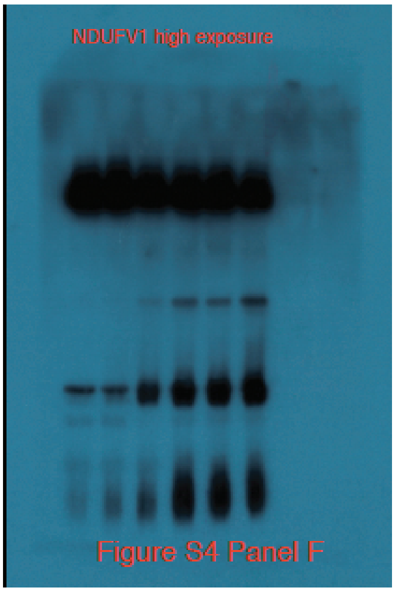

Supplementary Figure 6 Panel A

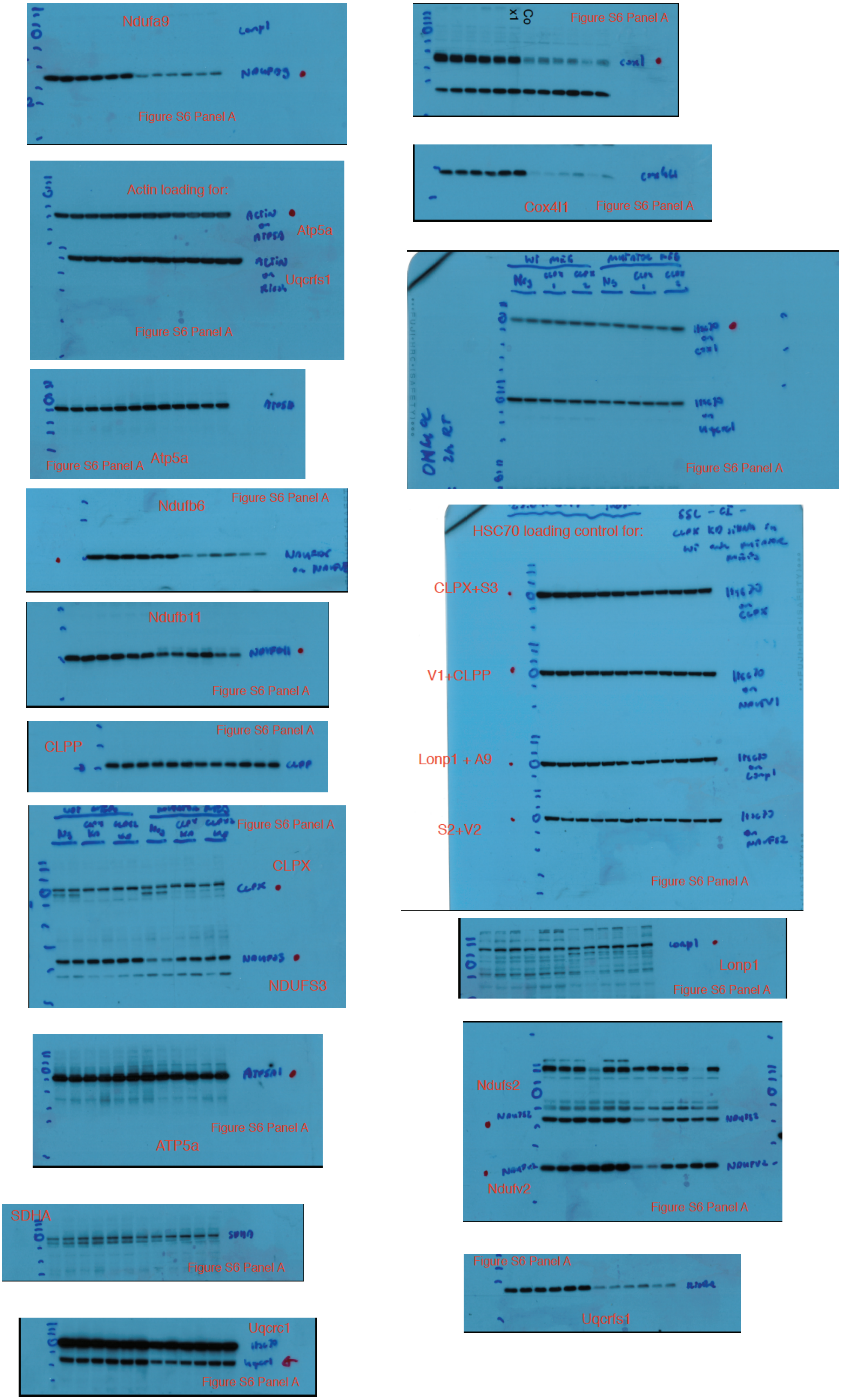

Supplementary Figure 6 Panel B

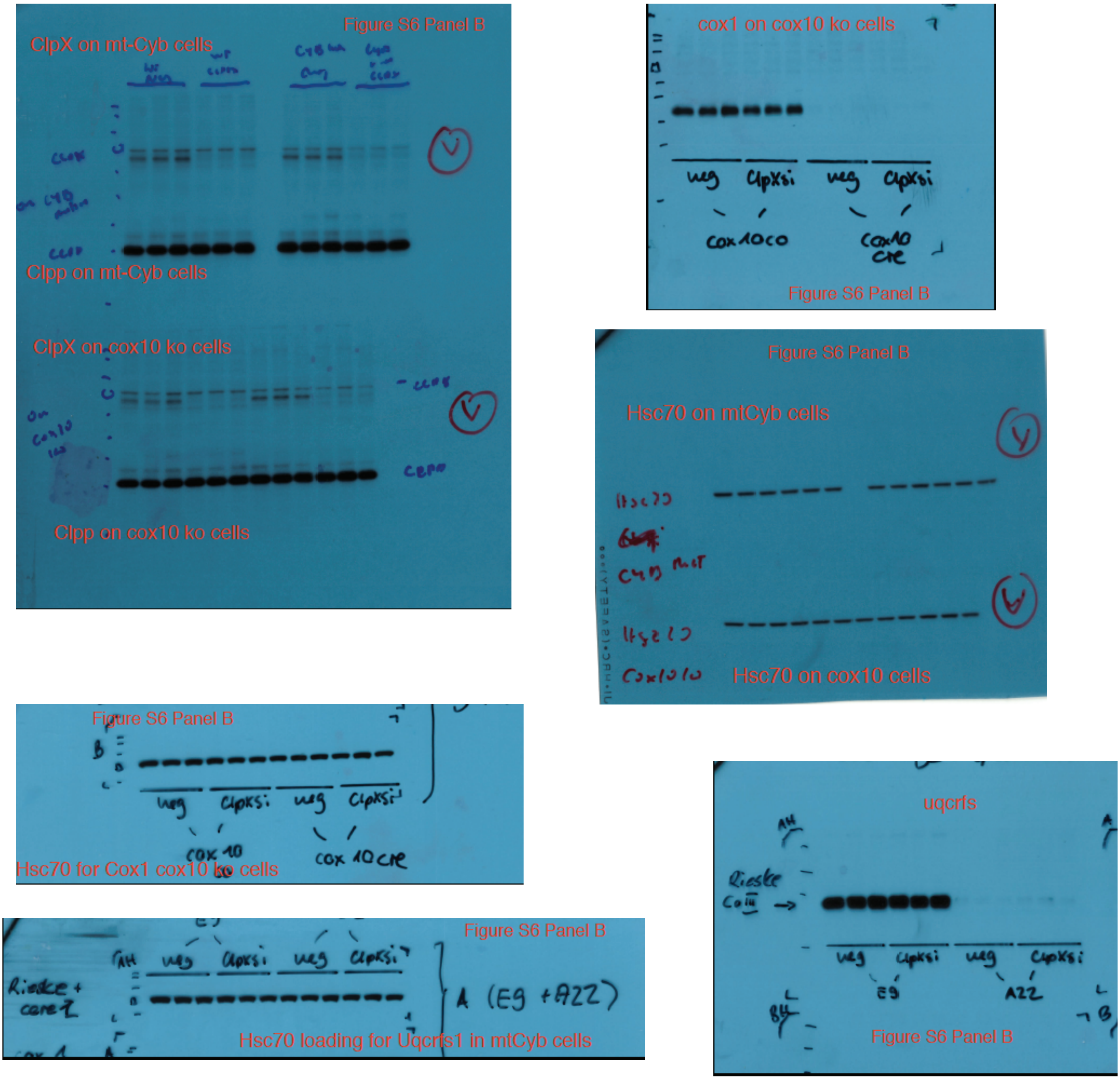

Supplementary Figure 6 Panel C

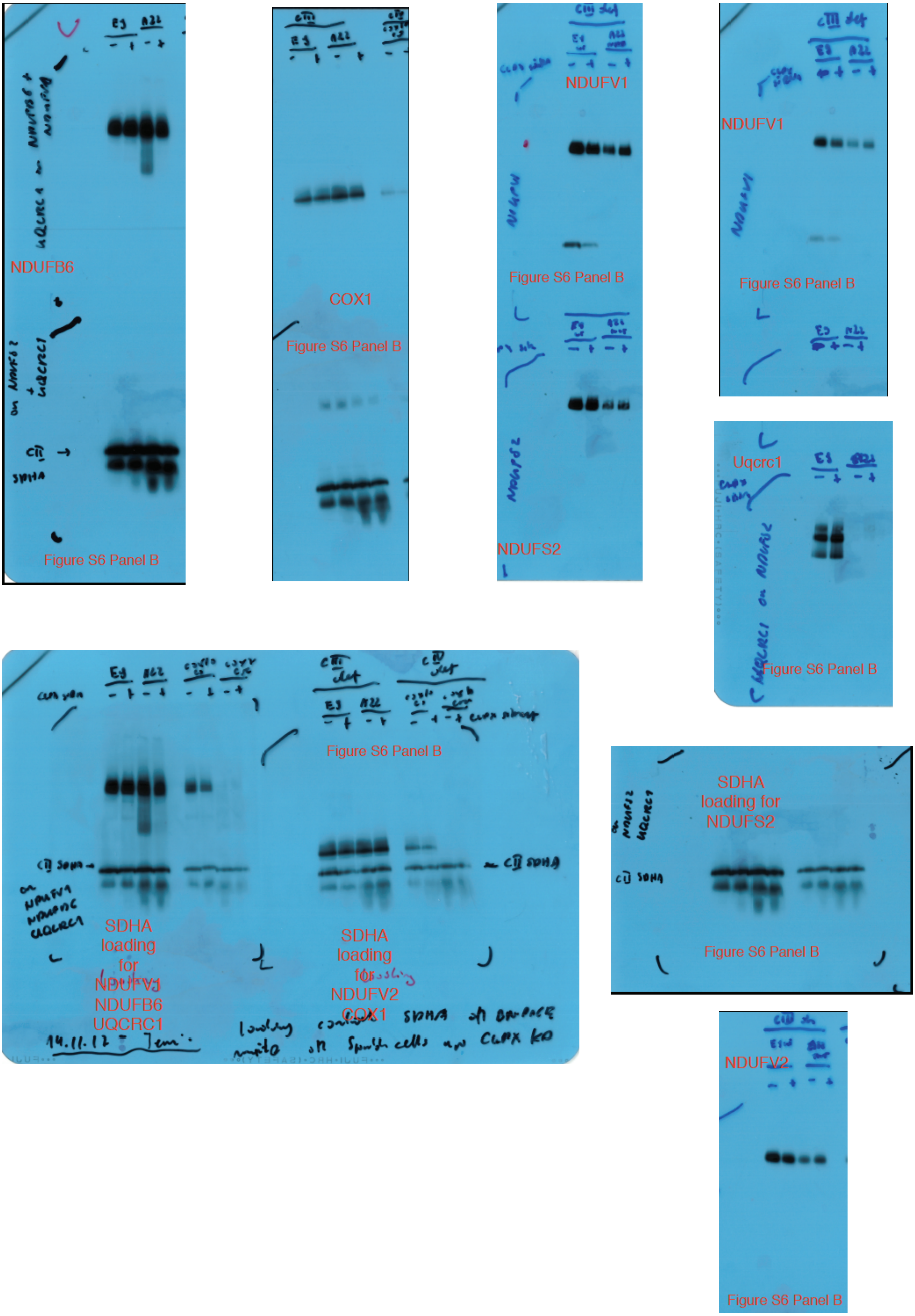

Supplement: Supplementary file 13 — Source Data [file 41467_2020_15467_MOESM13_ESM.pdf]
